# Potential Impacts of Offshore Wind Farms on North Sea Stratification

**DOI:** 10.1371/journal.pone.0160830

**Published:** 2016-08-11

**Authors:** Jeffrey R. Carpenter, Lucas Merckelbach, Ulrich Callies, Suzanna Clark, Lidia Gaslikova, Burkard Baschek

**Affiliations:** 1 Institute of Coastal Research, Helmholtz-Zentrum Geesthacht, Geesthacht, Germany; 2 Joint Program in Oceanography/Applied Ocean Science and Engineering, Massachusetts Institute of Technology, Woods Hole Oceanographic Institution, Woods Hole, MA, United States of America; University of Vigo, SPAIN

## Abstract

Advances in offshore wind farm (OWF) technology have recently led to their construction in coastal waters that are deep enough to be seasonally stratified. As tidal currents move past the OWF foundation structures they generate a turbulent wake that will contribute to a mixing of the stratified water column. In this study we show that the mixing generated in this way may have a significant impact on the large-scale stratification of the German Bight region of the North Sea. This region is chosen as the focus of this study since the planning of OWFs is particularly widespread. Using a combination of idealised modelling and *in situ* measurements, we provide order-of-magnitude estimates of two important time scales that are key to understanding the impacts of OWFs: (i) a mixing time scale, describing how long a complete mixing of the stratification takes, and (ii) an advective time scale, quantifying for how long a water parcel is expected to undergo enhanced wind farm mixing. The results are especially sensitive to both the drag coefficient and type of foundation structure, as well as the evolution of the pycnocline under enhanced mixing conditions—both of which are not well known. With these limitations in mind, the results show that OWFs could impact the large-scale stratification, but only when they occupy extensive shelf regions. They are expected to have very little impact on large-scale stratification at the current capacity in the North Sea, but the impact could be significant in future large-scale development scenarios.

## Introduction

**Fig 1:**
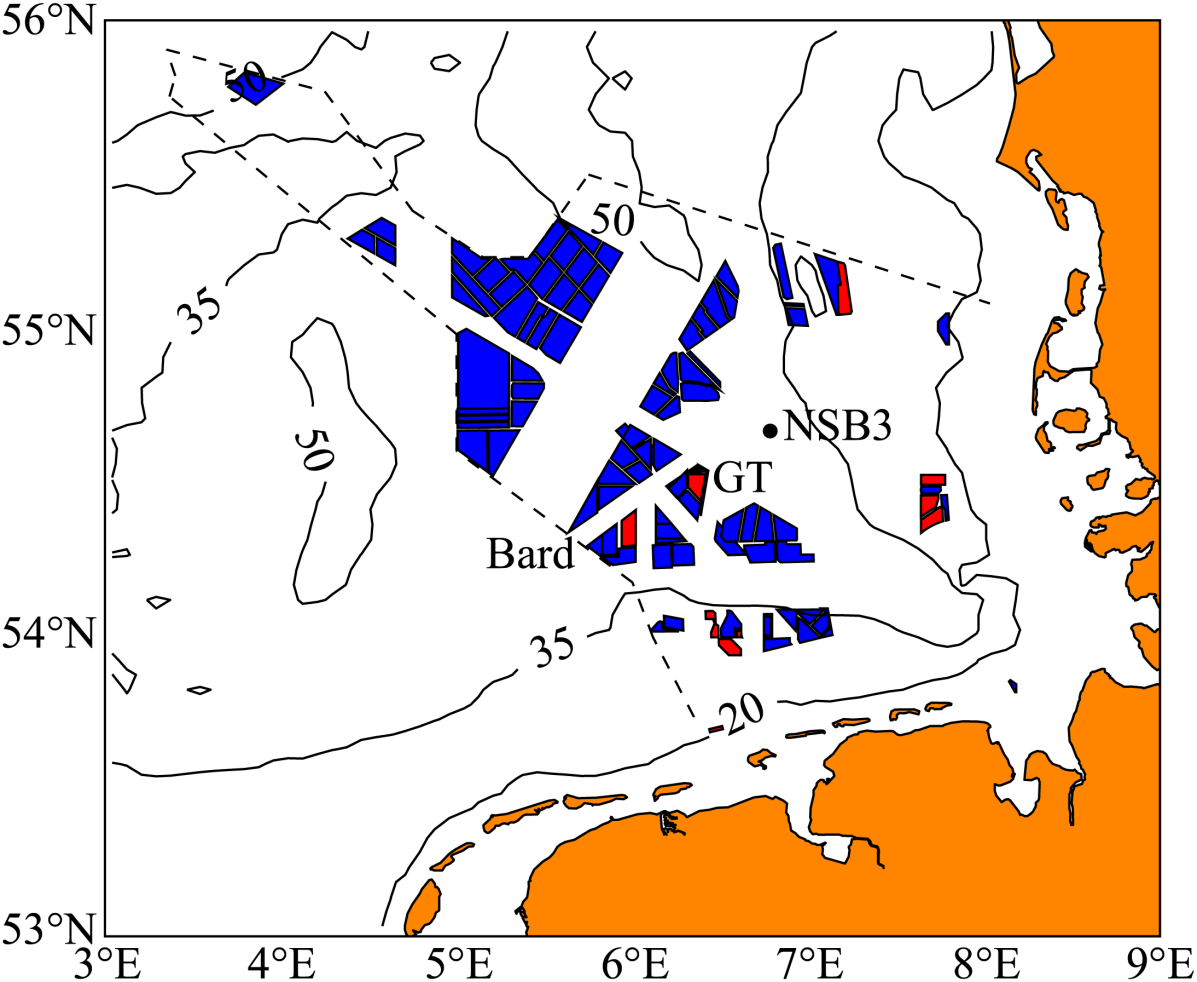
Map of wind farm developments in the German exclusive economic zone of the North Sea. Red denotes wind farms that are either operational or in construction, while blue denotes areas in the planning stage. The existing Bard and Global Tech (GT) farms are labelled, as well as the North Sea Buoy 3 (NSB3) measurement station. Contours are of mean water depth in m. Data obtained from the Bundesamt für Seeschifffahrt und Hydrographie (BSH).

**Fig 2:**
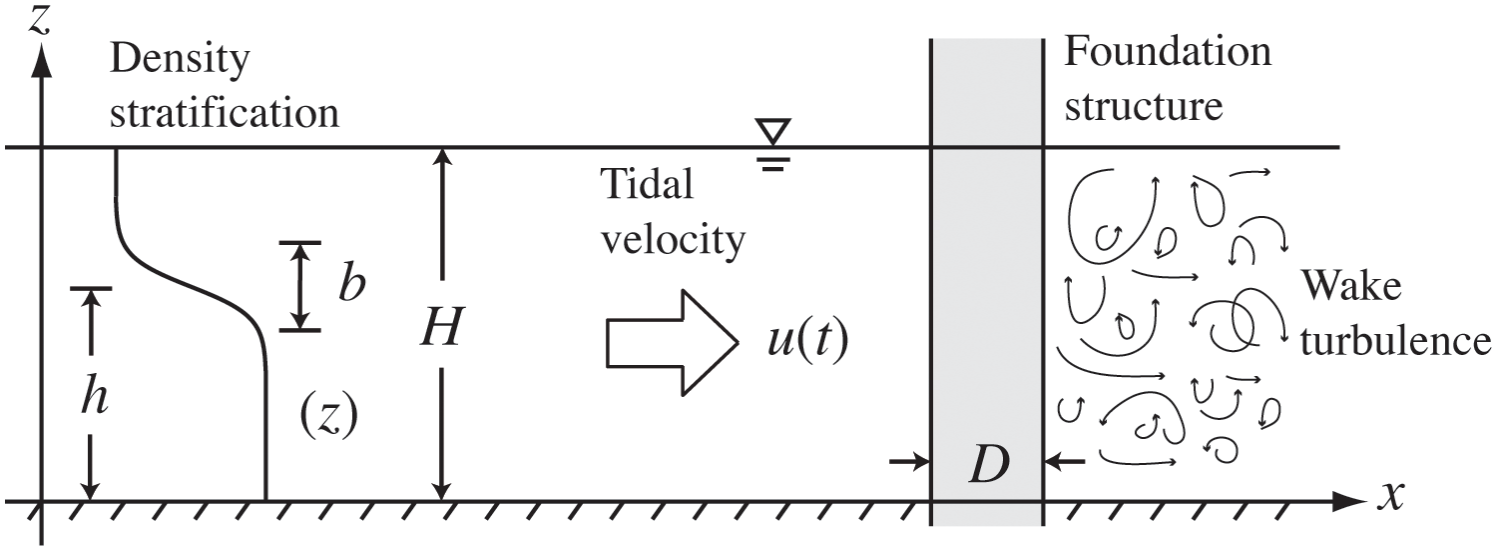
Basic sketch of the idealised setup considered. A typical density profile is illustrated where stratification (i.e., change in *ρ*(*z*)) is confined to a pycnocline layer with thickness, *b*, at height, *h*, from the sea bed. Only a single foundation structure is shown in the sketch.

In the search for sustainable, low-carbon, power production, many coastal regions are turning to offshore wind farming to meet rising energy demands. Through continuing technological development, offshore wind farms (OWFs) are now being built, or planned, in waters that are deep enough to be seasonally stratified. This is particularly true in the coastal regions of the German Bight sector of the North Sea, where a significant fraction of the total area could eventually be covered with wind farms (Fig 1). Such large-scale development raises a number of questions regarding the impacts of these structures on the coastal ocean environment. However, we focus on the possible large-scale impact that is caused by increased turbulent mixing of the seasonal stratification as the tides drive currents back and forth through the arrays of OWF structures (see diagram in Fig 2).

Stratification forms every summer in the North Sea when increased heat from solar radiation and increased air temperatures warm the upper layer of the water column, often creating temperature differences of 5 to 10°C from the cooler bottom layer. This stratification is only able to form in waters that are sufficiently deep, since both wind stress acting at the water surface, and bottom friction at the sea bed, are the primary sources of turbulence that act to destroy the stratification [1, 2]. The seasonal development of stratification, as well as its evolution in time through turbulent mixing, is known to have a large influence on the productivity and food web of the North Sea [3–5], with implications for carbon fixation [6], dissolved oxygen concentrations and hypoxia [7].

The literature on the impacts of OWFs is large, but has focussed to a great degree on marine life, e.g., birds [8, 9], sea mammals [10] and construction noise [11–13], and biofouling [14, 15]. The impacts span both negative and positive effects, such as the harmful impact of construction noise on mammalian behaviour [11], or the hypothesis that a single foundation structure could provide 2.5 times the amount of habitat that it destroys [16]. However, the hydrodynamic effects and related impacts on turbulence and mixing have not received nearly as much attention [17–19]. A closely related study was recently carried out by Rennau et al. [18] with respect to wind farm development in the Baltic Sea, where they focussed on impacts on the dense inflows to the Baltic from the North Sea. They found that a realistic OWF construction scenario, according to plans approved in 2010, could cause mixing in Baltic inflows and decrease bottom water salinity by 0.1 PSU. In an extreme scenario, with turbines filling the Danish Sounds, they calculated a 0.3 PSU decrease in bottom salinity, and a 2-metre shoaling of Baltic inflows. In that scenario wind farm generated mixing could cause the highest density inflows to disappear. It is important to note that the flow through the OWFs considered by Rennau et al. [18] is not caused by tidal currents, as we consider in this study.

The approach that we take in this study is highly idealised, and designed to understand whether or not it is possible for wind farms to generate enough mixing to significantly affect the stratification of the German sector of the North Sea. There are many uncertainties in our ability to parameterise structure-induced mixing, and the developed models are intended only as a series of order-of-magnitude estimates. Our principle goal is to bound and estimate two different time scales: (i) the mixing time scale, *τ*_mix_, that quantifies the residence time for stratification given a mixing rate (i.e., removal) that is characteristic of the OWF foundation structures, and (ii) the advective time scale, *τ*_adv_, that quantifies the residence time of a water parcel within a wind farm given the mean residual currents. From these two time scales we quantify the impact of the OWF structure mixing on the large-scale stratification.

The paper is organised as follows: idealised models for both turbulence production and the turbulent mixing of stratification are first developed, and then estimates of the mixing time scale are made with the use of a numerical model and different field measurements. A numerical model for residual currents is then used to estimate the advective time scale. Discussion and conclusions are presented in the final sections.

## Turbulence Production

We begin by developing a parameterisation for the power consumption of the wind farm foundations. Two wind farms currently built in deep (40 m) North Sea waters (Fig 1) that are often seasonally stratified are Bard 1 and Global Tech 1 (see [5], and later discussion). Both have foundation structures that consist of a number of cylindrical sections that are mounted to the sea floor. The Bard 1 “tripile” foundation consists of three cylinders mounted vertically, however, the Global Tech 1 “tripod” foundation is more complex and described in detail in the supplementary information. These foundation structures are modelled by assuming the idealised situation of a group of cylinders in a tidally oscillating cross flow (see the diagram in Fig 2). The following formula gives the drag force that a cylinder exerts on the passing (tidal) flow,

$$\begin{array}{c} \vec{F}=-\frac{1}{2}\rho_{0}C_{D}A\left|\vec{u}\right|\vec{u}, \end{array}$$ (1)

where *ρ*_0_ is the density of the fluid, *C*_*D*_ is the drag coefficient, *A* is the frontal area of the cylinder that is exposed to the free stream, with *A* = 3*DH* for the Bard tripile foundation where *D* is the diameter, and *H* the total water depth, and $\vec{u}$ is the velocity of the free stream.

Since we are interested in time scales longer than the dominant tidal periods (semidiurnal periods M_2_ and S_2_ are dominant in the German Bight [20]), we assume that the water depth, *H*, is constant. In addition, despite the major role that differences in water density will play in this paper, in the above formula we shall take the water density to be constant, represented by the naught subscript on *ρ*_0_. This is justified because the changes in water density caused by stratification, Δ*ρ*, are much less than the mean density, i.e., Δ*ρ*/*ρ*_0_ ≪ 1, and so have little affect on the cylinder drag formula.

The value of *C*_*D*_ is known to depend on a number of parameters, and is one of the most important sources of uncertainty in our parameterisation of foundation structure turbulence. *C*_*D*_ has been found to depend on the Reynold’s number $\text{Re}\equiv |\vec{u}|D/\nu$, the relative roughness *κ*/*D*, with *ν* the kinematic viscosity of the fluid and *κ* a measure of the roughness length scale, as well as on the amount of turbulence in the free-stream approach flow [21]. To retain this uncertainty in our analysis, we shall use two estimates of *C*_*D*_ in this study that span the expected range, *C*_*D*_ = 0.35, 1.0, which we refer to as the low- and high-drag cases, respectively. Note that Rennau et al. [18] use a *C*_*D*_ = 0.63 in their study of OWF induced mixing in the Baltic Sea. Estimates of all these parameters are listed in Table 1, with details of our estimates of OWF structure parameters outlined in S1 File in the supporting information.

**Table 1:** Summary of representative values for wind farm and stratification parameters.

	Structure Parameters				Water Column Parameters					
Wind Farm	*C*_*D*_	*A* ^†^	*ℓ*^†^	*P*_str_	*ρ*_0_	*H*	*h* ^‡^	*b* ^‡^	Δ*ρ* ^‡^	$\phi_{\text{max}}^{‡}$
	(-)	(m^2^)	(m)	(mW m^−2^)	(kg m^−3^)	(m)	(m)	(m)	(kg m^−3^)	(kJ m^−2^)
Bard 1	0.35, 1.0	402	866	**3.3**, 9.4	1026	40	28	6	3.1	5.0
Global Tech 1	0.35, 1.0	560	733	5.3, **15**	1026	40	28	6	3.1	5.0

^†^Estimates of frontal areas and mean turbine spacings for each of the farm foundation structures are outlined in the supporting information.

^‡^These water column parameters are taken to be representative of the peak of summer stratification, as seen from the observations from 2014 (for *h* and *b*) or both 2009 and 2014 (as for Δ*ρ* and *ϕ*_max_). These observations are discussed later in the paper.

Because the force is parallel to the velocity, and the power is given by $\vec{F}\cdot \vec{u}$, we can write the power removed from the flow by the cylinder as

$$\begin{array}{c} \text{Power}\;\text{removed}=\frac{1}{2}\rho_{0}C_{D}A{|\vec{u}|}^{3}. \end{array}$$ (2)

However, it is more convenient to express the power consumption per unit area, so our formula will now apply within a wind farm, providing a mean value, and is given by

$$\begin{array}{c} P_{\text{str}}=\frac{\rho_{0}C_{D}A\left\langle {|\vec{u}|}^{3}\right\rangle }{2\ell^{2}}, \end{array}$$ (3)

where *P*_str_, in W m^−2^, is the power removed by the structures from the flow per unit area of wind farm, with *ℓ* the distance between equally spaced wind turbines in a much larger farm, and $\langle {|\vec{u}|}^{3}\rangle$ represents the mean cubed current velocity averaged over a period much longer than the dominant tidal periods. This estimate of the power removed by the wind farm foundation structures is assumed to be equal to the power put into the turbulence production in order to calculate mixing rates, as described in the following section.

Using the wind farm parameters listed in Table 1, all that is needed to calculate *P*_str_ are mean cubed tidal current velocities. These were obtained from using a two-dimensional version of the TRIM-NP hydrodynamic model [22, 23]. Barotropic currents were calculated in the North Sea and part of the north eastern Atlantic Ocean using a regular grid of 12.8 km, with a finer nested grid of 1.6 km resolution in the German Bight region. The tidal currents and surface elevations are prescribed at the open lateral boundaries using the FES dataset [24], and the atmospheric forcing is provided from the regional COSMO-CLM historical simulation [25]. Simulations for the period of Jan. 1948 to Aug. 2015 are freely available (doi: 10.1594/WDCC/coastDat-2_TRIM-NP-2d). The results of the TRIM model for the calculation of $\langle {|\vec{u}|}^{3}\rangle$ were found to be within ±5% of the values using the Copernicus Marine Environmental Monitoring Service data set (http://marine.copernicus.eu) for the Northwest European Shelf Seas region for the Bard and Global Tech OWFs. Additional validation of the model is discussed in Weisse et al. [26].

**Fig 3:**
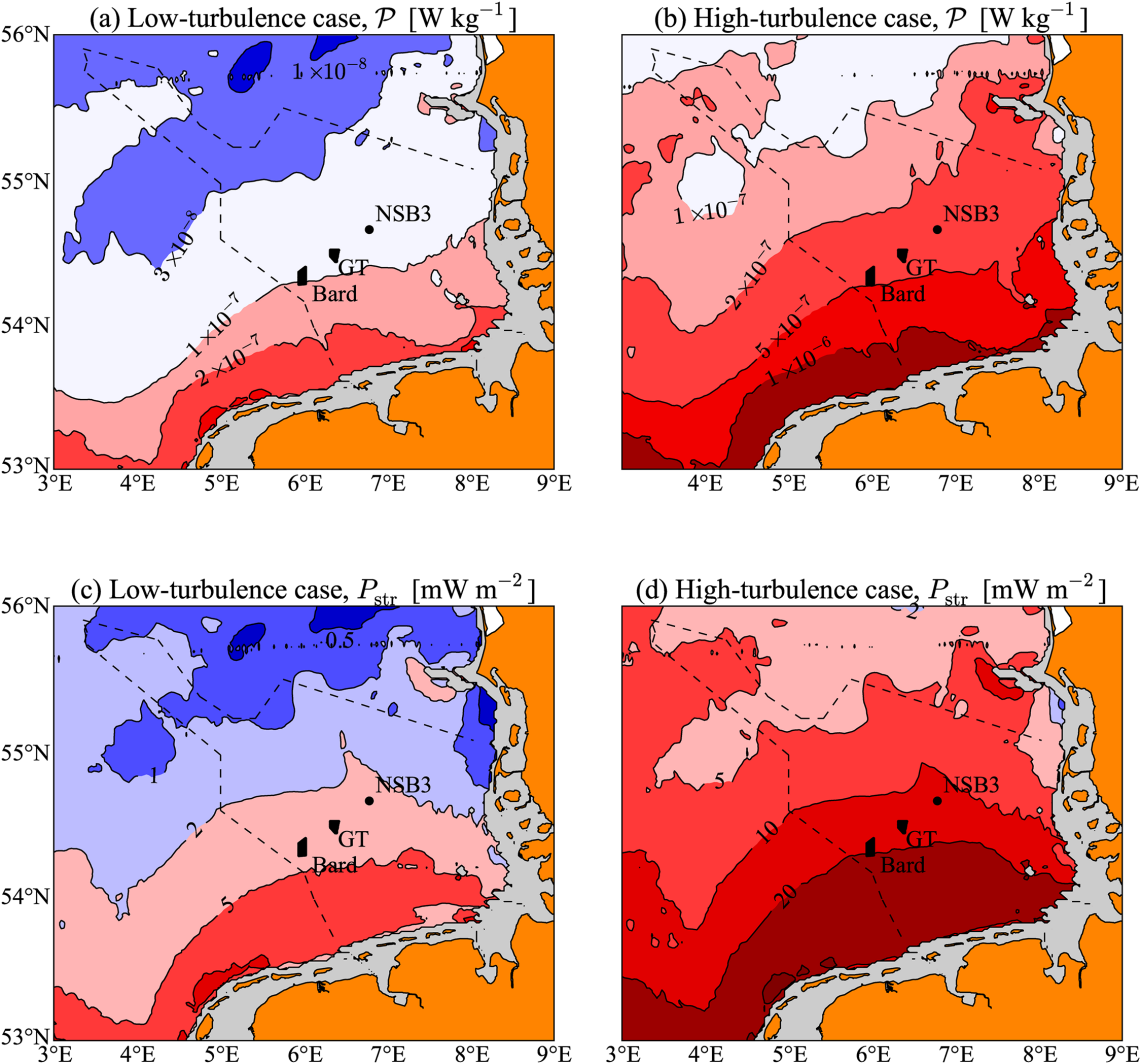
Map of estimated potential wind farm induced turbulence production in the German Bight region of the North Sea. Contours and colours show $P=P_{\text{str}}/\left(\rho_{0}H\right)$ in units of W kg^−1^ (a, b) and *P*_str_ in mW m^−2^ (c, d). We have shown both low- and high-turbulence cases for the Bard 1 parameters with *C*_*D*_ = 0.35 in (a, c), and Global Tech 1 parameters with *C*_*D*_ = 1.0 in (b, d). The spatial pattern in (a, b) reflects the modelled distribution of $\langle {|\vec{u}|}^{3}\rangle$, and the panels differ only by a factor of 4.6 (the difference in wind farm parameters for the high- and low-turbulence cases). The location of both the Bard 1 and Global Tech 1 (GT) farms are shown, along with the North Sea Buoy 3 (NSB3) measurement station. Grey areas are not included because they are on average less than 10 m deep, or comprise tidal flats or estuaries.

This estimate of $\langle {|\vec{u}|}^{3}\rangle$ allows us to map *P*_str_ for the North Sea, as shown in Fig 3(c) and 3(d). The quantity $P\equiv P_{\text{str}}/\left(\rho_{0}H\right)$ is also plotted in Fig 3(a) and 3(b), giving an estimate of the average power that could potentially be delivered to the turbulence by the OWF foundations in any region of the German Bight per unit mass of water, with units W kg^−1^. Two extreme cases are shown in Fig 3, and these correspond to (a, c) the low-turbulence case where we choose the low drag coefficient and the Bard foundation structures, and (b, d) the high-turbulence case where we choose the high drag coefficient and the Global Tech foundation structures. The difference between the low- and high-turbulence cases is a factor of 4.6, demonstrating that there are significant uncertainties in the amount of OWF structure induced turbulence. The maps of Fig 3 show that $P,P_{\text{str}}$ exhibit large regional variation within the German Bight, and generally decreases northwards with distance from the coast, which follows from the fact that the tidal velocities are strongest in these shallower regions. The large changes in $P,P_{\text{str}}$ shown in each of the low-turbulence panels of Fig 3(a) and 3(c) are, however, likely to be an overestimate. This is due to the dependence of *C*_*D*_ on the current velocity. Our low-drag value of *C*_*D*_ = 0.35 is chosen to be representative of circular cylinders that have surpassed the drag crisis, i.e., a sudden drop in *C*_*D*_ that occurs at $\text{Re}≳3\times 10^{6}$. As the tidal currents reduce away from the coasts, we should therefore see a higher likelihood of the high-drag scenario. Despite this effect, we can generally expect lower $P,P_{\text{str}}$ in areas further offshore.

Values for *P*_str_ in the Global Tech and Bard 1 farms for both the high- and low-drag cases are shown in Table 1. We find an approximate range that is bounded by 3.3 and 15 mW m^−2^ for the average rate of turbulence production for these two farms. These two extreme values will be used throughout the rest of the paper, and shall be referred to as the high- and low-turbulence cases.

Note that since *P*_str_ depends on the cube of the tidal velocities, it is relatively sensitive to errors in $|\vec{u}|$. We have compared the estimates of *P*_str_ from the TRIM model with measurements using an Acoustic Doppler Current Profiler (ADCP) at the North Sea Buoy 3 (NSB3) station, taken over a 45 day period from mid-July to the end of August in 2014 (to be discussed in the observations section). The comparison shows only a 22% difference in the value of *P*_str_ calculated from the ADCP measurements compared to the model results (with model results yielding larger values). This discrepancy is quite small considering the other uncertainties, e.g., *C*_*D*_ and foundation structure type, that lead to a factor of 4.6 between the high- and low-turbulence estimates.

To put the calculated turbulence levels in perspective, it is interesting to compare the turbulence production by the turbine structures, *P*_str_, with that occurring in the boundary layer at the sea bed. The latter can be quantified by

$$\begin{array}{c} P_{\text{bot}}=\rho_{0}C_{D}^{\text{bot}}{|\vec{u}|}^{3}, \end{array}$$ (4)

where the bottom drag coefficient $C_{D}^{\text{bot}}\approx 2.5\times 10^{-3}$ [27]. Taking the ratio leads to

$$\begin{array}{c} \frac{P_{\text{str}}}{P_{\text{bot}}}=\frac{C_{D}A}{2C_{D}^{\text{bot}}\ell^{2}}, \end{array}$$ (5)

and when we substitute typical values we find that despite the much larger OWF structure drag coefficient $C_{D}/C_{D}^{\text{bot}}=140-400$, the equivalent area of the structures relative to the total sea floor area *A*/*ℓ*^2^ ≈ 0.5 − 1.0 × 10^−3^ is much smaller, and results in a relative power consumption of *P*_str_/*P*_bot_ ≈ 0.037 − 0.21. This implies that the total power that is extracted from the tides by the turbine structures is approximately 4 − 20% of the bottom boundary layer extraction. As farms are built in deeper waters this percentage will increase linearly with the water depth, *H*. This suggests that the OWF-induced turbulence could be significant compared to the natural sources. In addition, the OWF turbulence will be distributed throughout the water column, whereas the bottom boundary layer turbulence is mainly confined to the near-bed region [27].

## Mixing of Stratification: Idealised Modelling

Using the estimate of the power removal by the wind farm foundations that was developed above, we now examine the potential impact on the North Sea density stratification. The basic assumption is that the power removed from the free-stream tidal currents is converted into small-scale turbulent motions. These motions then dissipate their energy through both internal friction (i.e., viscous dissipation) and by a mixing of the water column stratification. In this section we provide a series of idealised estimates of this mixing in the North Sea by different methods under the assumption that no other turbulent mixing processes are acting simultaneously. Our main goal is to estimate characteristic residence time scales for the stratification, which can be compared with the time of seasonal stratification build up, and an advective time scale quantifying the residence time of water within an OWF.

The general approach we take to estimating the turbulent mixing of the wind farm foundations is to satisfy the conservation of energy for the turbulent flow. As a first approximation we shall neglect horizontal advection by the large-scale residual currents (i.e., averaged over periods much larger than the dominant tidal periods). The effect of residual currents is discussed in a later section. Neglecting this residual advection for the moment, we can write an evolution equation in time, *t*, for the mean density profile, *ρ*(*z*, *t*), within the wind farm as

$$\begin{array}{c} \frac{\partial \rho }{\partial t}=\frac{\partial }{\partial z}(\frac{\rho_{0}}{g}B), \end{array}$$ (6)

where *g* is gravitational acceleration, and *B* is the vertical buoyancy flux due to turbulent mixing acting on the density field. The sign convention of *B* < 0 for the mixing (i.e., destruction) of the stratification is used. In turbulent mixing studies the buoyancy flux is often written explicitly as $B=-g\;\bar{w^{\prime }\rho^{\prime }}/\rho_{0}$, where the primes denote values associated with turbulent eddies, and the overbar represents an ensemble average over many turbulent events. We can derive an equation for a measure of the potential energy of the water column, *ϕ*(*t*), defined by

$$\begin{array}{c} \phi \left(t\right)\equiv \int_{0}^{H}\left[\rho_{\text{mix}}-\rho \left(z,t\right)\right]gz\;dz, \end{array}$$ (7)

where *ρ*_mix_ is the density if the entire water column were completely mixed. Note that the presence of *ρ*_mix_ only constitutes a choice of datum with respect to which the potential energy is measured, and that *ϕ* really represents the amount of energy required to mix the water column. Throughout the rest of this paper we refer to *ϕ* as the “stratification”. A mixing of the water column, caused by *B* < 0, corresponds to a decrease in *ϕ* over time. This can be seen by forming an evolution equation for *ϕ*, obtained directly from Eq (6) to give

$$\begin{array}{c} \frac{d\phi }{dt}=\rho_{0}\int_{0}^{H}B\;dz, \end{array}$$ (8)

where we have assumed that there are no fluxes of buoyancy (and potential energy) across the boundaries at *z* = 0, *H*.

In the often-used eddy diffusivity formulation of turbulent mixing, *B* is parameterised by the diffusive law

$$\begin{array}{c} B=-KN^{2}\;\text{with}\;N^{2}\equiv -\frac{g}{\rho_{0}}\frac{\partial \rho }{\partial z}, \end{array}$$ (9)

with *K*(*z*, *t*) the turbulent diffusivity. This parameterisation results in a diffusion equation for *ρ* with variable diffusivity, i.e.,

$$\begin{array}{c} \frac{\partial \rho }{\partial t}=\frac{\partial }{\partial z}(K\frac{\partial \rho }{\partial z}). \end{array}$$ (10)

In order to connect the stratification and the turbulence we use a conservation equation for the kinetic energy of the turbulence that consists of a balance between three terms: (i) the production of turbulent kinetic energy, P, (ii) the rate at which this energy performs work on the buoyancy field (i.e., mixing stratificaton), *B*, and (iii) the rate at which the energy is lost to viscous dissipation (i.e., frictional losses), *ε*. This three-way balance is written

$$\begin{array}{c} P+B-\varepsilon =0, \end{array}$$ (11)

and is often referred to as the local equilibrium hypothesis. In general, the production term P may have a number of different contributions, one of which is through the turbulence generation of the turbine structures (Fig 3). In this case, we have $P=P_{\text{str}}/\rho_{0}H$, which is independent of time, since we are dealing with time scales longer than the dominant tidal periods, and assumed to be independent of *z*.

Using the basic equations above, we now describe three idealised mixing models (i.e., parameterisations) of increasing complexity in order to estimate a characteristic residence time for the stratification, *τ*_mix_.

### Model 1: Constant pycnocline thickness

In the first instance we take the density profile to be represented by a pycnocline region, to which the density stratification is confined, with a thickness *b*, and a centre that is located at the height *h*, above the seabed (Fig 2). Outside of the pycnocline the upper and lower layers are completely mixed so that the stratification there is negligible. We also assume that in the pycnocline there is a constant partitioning of the energy production by the turbine structures, P, and energy used to perform work on the buoyancy field, *B*. In other words, we assume the constant mixing efficiency, or flux Richardson number

$$\begin{array}{c} R_{f}\equiv -B/P, \end{array}$$ (12)

with the value of *R*_*f*_ = 0.17 often used in oceanographic studies [28]. This constant mixing efficiency relation can be used to find the rate of decrease in the stratification due to mixing from Eq (8) of

$$\begin{array}{c} \frac{d\phi }{dt}=-R_{f}\frac{b}{H}P_{\text{str}}. \end{array}$$ (13)

This simple equation is our first estimate of the mixing induced by the wind farm foundations. It can be interpreted as a constant fraction of the turbulence supplied by the foundation structures being used to mix the stratification, and mixing occurs only within the stratified region of the water column (i.e., within the pycnocline), hence the factor *b*/*H*. This fraction is easily seen to be related to a bulk mixing efficiency defined by

$$\begin{array}{c} \left\langle R_{f}\right\rangle \equiv -\int_{0}^{H}B\;dz/\int_{0}^{H}P\;dz=R_{f}\frac{b}{H}. \end{array}$$ (14)

It is now of interest to use Eq (13) to calculate an approximate residence time of the stratification. This is done by the following general formula,

$$\begin{array}{c} \tau \equiv -\frac{\phi }{d\phi /dt}\;\Rightarrow \;\tau_{\text{mix}}=\frac{\phi_{\text{max}}H}{R_{f}P_{\text{str}}b}. \end{array}$$ (15)

Using the typical values given in Table 1 leads to a residence time of *τ*_mix_ = 688 days and 151 days for the low- and high-turbulence cases, respectively. Note that this estimate is based on a constant interface thickness of *b* = 6 m, and assumes that the mixing induced by the wind farm foundations does not result in a change of the pycnocline in time. It can therefore be thought of as an approximate lower bound on the mixing. On the other hand an extreme upper bound on the mixing can be found by taking *b* = *H*, giving a residence time of *τ*_mix_ = 103 days and 23 days, respectively. A more accurate upper bound will be developed in the next subsection that accounts for the growth of the pycnocline in time. However, we can see that there is a significant difference in our two extreme estimates, and the results will depend heavily on the evolution of the pycnocline, which largely determines the bulk mixing efficiency, 〈*R*_*f*_〉.

### Model 2: A time-dependent pycnocline model

In this model we attempt to account for a time-dependent pycnocline thickness. In contrast to the last section, we choose a well-defined continuous error function (erf) representation given by

$$\begin{array}{c} \rho \left(z,t\right)=\rho_{0}-\frac{\Delta \rho }{2}\text{erf}[\frac{\sqrt{\pi }\left(z-h\right)}{b(t)}], \end{array}$$ (16)

where *h* is the height of the pycnocline, Δ*ρ* the density difference across the pycnocline, and *b*(*t*) is a measure of its thickness, which we now assume is increasing in time due to mixing. An increasing pycnocline thickness with time has been observed in the laboratory experiments of Whitehead [29], where a cylindrical rod was used to produce turbulence by stirring water with a two-layer salt stratification in a tank. Whitehead [29] found that the thickening pycnocline resulted when the stratification was relatively weak (as measured by an appropriately defined Richardson number). The error function profile used above is a well known solution to the diffusion equation and also resembles the solutions found in the Whitehead [29] experiments.

Using this continuous profile, *b* is defined more precisely as

$$\begin{array}{c} b\equiv \frac{\Delta \rho }{{\left(\partial \rho /\partial z\right)}_{\text{max}}}. \end{array}$$ (17)

The vertical coordinate has the range 0 < *z* < *H*, with *z* = 0 the sea bed, and *z* = *H* the water surface. At the moment we will ignore the fact that this profile does not satisfy the appropriate no-flux boundary conditions at *z* = 0, *H*, and limit ourselves to values of *b* that do not exceed the depth of the pycnocline, i.e., times for which *b* < *min*{*h*, *H* − *h*}. We therefore cannot use this method as such to precisely calculate the time to complete mixing. By treating Δ*ρ* as a constant, but allowing *b* to vary in time, we can however, determine the rate of change of *ϕ* in time due to a thickening pycnocline as

$$\begin{array}{c} \frac{d\phi }{dt}=\int_{0}^{H}\frac{\partial }{\partial t}\rho \left(z,t\right)gz\;dz. \end{array}$$ (18)

Performing the derivative, the change in *ϕ* can be written as

$$\begin{array}{c} \frac{d\phi }{dt}=\frac{g\Delta \rho }{b^{2}}f\left(b\right)\frac{db}{dt}, \end{array}$$ (19)

where

$$\begin{array}{c} f\left(b\right)\equiv \int_{0}^{H}z\left(z-h\right)\text{exp}[-\frac{\pi {\left(z-h\right)}^{2}}{b{\left(t\right)}^{2}}]dz. \end{array}$$ (20)

Evaluation of this integral can be shown to result in the relation *f*(*b*) = *b*^3^/2*π*.

We can now use this to equate the rate of change of *ϕ* in Eq (19) with the amount of power that is put into mixing from Eq (13), and rearrange to get the following simple relationship for the (constant) rate of increase in interface thickness

$$\begin{array}{c} \frac{db}{dt}=\frac{2\pi R_{f}P_{\text{str}}}{g\Delta \rho H}. \end{array}$$ (21)

Again using the typical values from Table 1, gives a rate of interface thickening of 0.25 and 1.1 m day^−1^ for the low- and high-turbulence cases, respectively. Note that as a quick estimate of the mixing time we can calculate how long it takes for the interface to thicken to the water depth, *H*/(*db*/*dt*) = 160 and 35 days. This analysis highlights how important it is to account for the thickness of the pycnocline in our evaluation of the total power that is put into turbulent mixing.

#### Accounting for boundaries

We shall now extend this model to account for the appropriate no-flux boundary conditions to model the evolution of *ρ*(*z*, *t*) until the time of complete mixing. This model is essentially equivalent to a solution of the diffusion Eq (10), with a diffusivity, *K*(*t*), that is a function only of *t*.

We begin with the following initial condition of the erf-type

$$\begin{array}{c} \rho \left(z,t=0\right)=\rho_{0}-\frac{\Delta \rho }{2}\text{erf}[\frac{\sqrt{\pi }\left(z-h\right)}{b_{0}}] \end{array}$$ (22)

with *b*_0_, *h*, *H*, *ρ*_0_ and Δ*ρ* all fixed by the initial condition, and given by the values in Table 1. However, in order to satisfy no-flux boundary conditions of

$$\begin{array}{c} -K\frac{\partial \rho }{\partial z}=0\;\text{at}\;z=0,H, \end{array}$$ (23)

we must add an infinite number of additional images [30], identical to our profile, but whose pycnocline lies outside the domain, i.e., we let

$$\begin{array}{c} \rho \left(z,t\right)=\rho_{0}+\frac{\Delta \rho }{2}\sum_{n=-\infty }^{+\infty }\{\text{erf}[\frac{\sqrt{\pi }\left(z+h+2nH\right)}{b(t)}]-\text{erf}[\frac{\sqrt{\pi }\left(z-h+2nH\right)}{b(t)}]\}. \end{array}$$ (24)

In practice, however, we have found that 6 images are sufficient to satisfy the boundary conditions and conserve mass. The evolution of *ρ*(*z*, *t*) proceeds while ensuring that Eq (13) is satisfied at all times.

**Fig 4:**
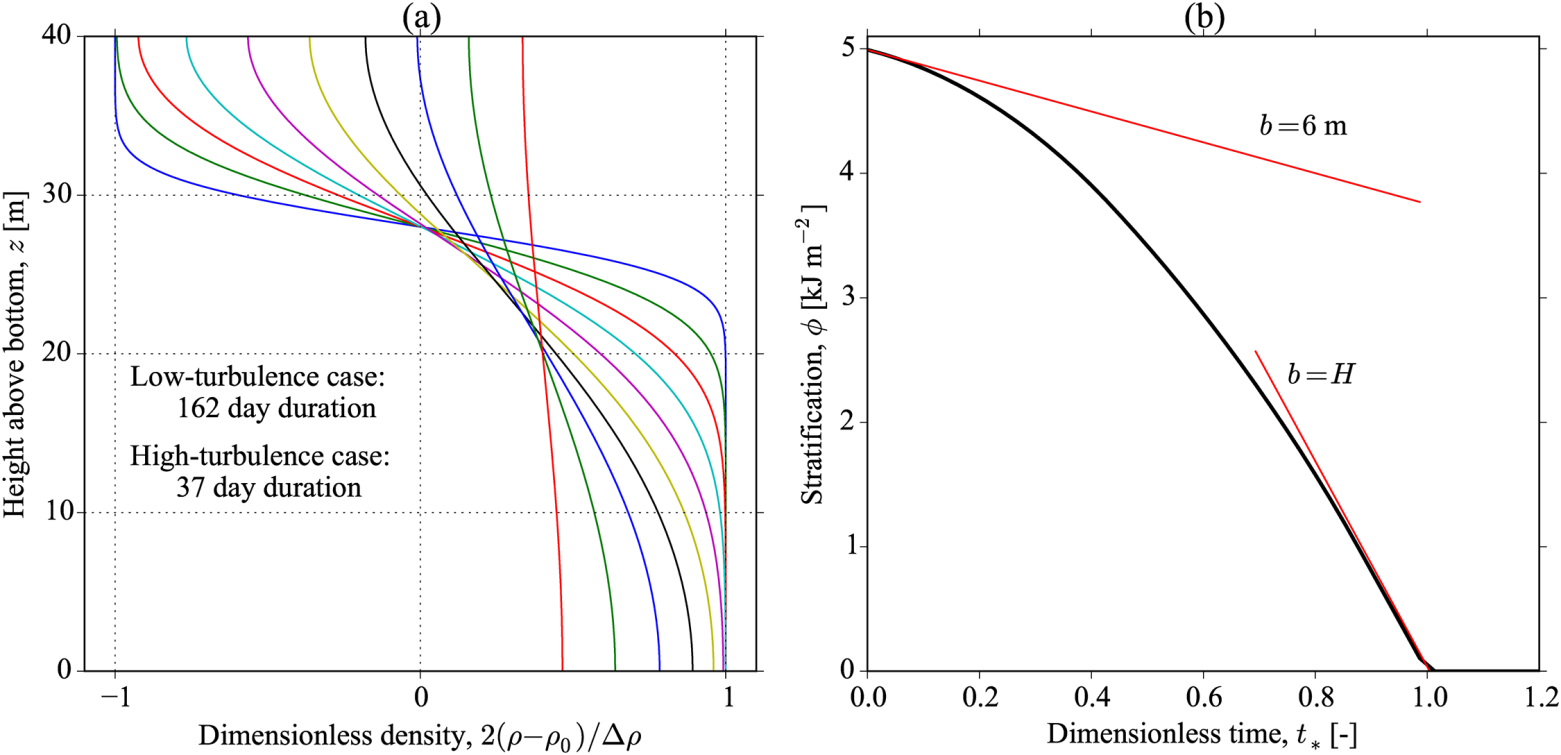
Solution for the time-dependent pycnocline model 2. The results of both low- and high-turbulence simulations are identical but span different time periods, since the solutions collapse if the dimensionless time t_*_ is used. The evolution of the dimensionless density profile 2(*ρ* − *ρ*_0_)/Δ*ρ* is shown in (a), along with the decrease in the stratification over time in (b). Also shown in (b) are the constant pycnocline thickness model mixing rates given by Eq (13) with *b* = 6 m and *b* = *H* = 40 m for comparison (slopes of red lines). In the low- and high-turbulence cases of (a), density profiles are identical, and plotted every 18 and 4 days, respectively.

The solution is shown in Fig 4(a) for both low- and high-turbulence cases, which show a collapse when plotted using the dimensionless time defined by t_*_ ≡ *tP*_str_/(*g*Δ*ρH*^2^). This scaling of the time variable is suggested by Eq (21), and the time that it takes the interface to thicken to the full water depth, i.e., *H*/(*db*/*dt*). The time to complete mixing can be defined by this dimensionless time variable, and through inspection of Fig 4(b) we choose *τ*_mix_ ≡ *g*Δ*ρH*^2^/*P*_str_, which corresponds to t_*_ = 1.0. This choice leads to mixing time scales of 170, and 38 days (the approximate duration of the plots shown in Fig 4a), for the low- and high-turbulence cases. Also plotted in Fig 4(b) are the constant pycnocline predictions for the fixed values of *b* = 6 m and *b* = *H* = 40 m (red lines).

Despite the support for a model of this type from the experiments of Whitehead [29], in which the pycnocline thickens in time due to a source of turbulence that is constant in depth, in reality the interface will eventually grow until it is influenced by the turbulence of the upper mixed layer, and the bottom boundary layer. After this time, the model should not be considered to be valid, since these sources will tend to sharpen the pycnocline and decrease *b*. Nonetheless, this mixing model shows that the mixing time scale is sensitive to the evolution of the pycnocline.

### Model 3: A one-dimensional mixing model

In this section we shall formulate a simple one-dimensional (1D) mixing model to account for the vertical structure of turbulent mixing. The goal is, once again, to determine a representative mixing time for the turbine structures to destroy the summer stratification, assuming that no other mixing processes are occurring. We use the diffusive model for the density fluxes from Eq (10) to determine the density profile over time, where *K* = *K*(*z*, *t*) now. In addition, we use the local equilibrium form for the turbulent kinetic energy as in Eq (11) with the production term $P=P_{\text{str}}/\left(\rho_{0}H\right)$, and the buoyancy flux term *B* = −*KN*^2^, i.e.,

$$\begin{array}{c} P_{\text{str}}/\rho_{0}H-KN^{2}-\varepsilon =0. \end{array}$$ (25)

Once again P is taken independent of *t*, and is assumed independent of *z*. It remains to provide parameterisations for both *K* and *ε*.

Following successful 1D turbulence modelling in the Irish Sea by Simpson et al. [27], we shall begin by using a similar approach. The dissipation of turbulent kinetic energy, *ε* (in m^2^ s^−3^, or equivalently W kg^−1^), shall be modelled by the relation

$$\begin{array}{c} \varepsilon =C_{1}\frac{q^{3}}{l}, \end{array}$$ (26)

where *C*_1_ is an empirical constant (see Table 2 for a summary of constants), *l* is the turbulent length scale which must be modelled, and we have chosen to use the turbulent velocity scale $q=\sqrt{2k}$ instead of the turbulent kinetic energy, *k*. In addition, the diffusivity shall be given by

$$\begin{array}{c} K=\frac{C_{2}}{\sqrt{2}}ql, \end{array}$$ (27)

where *C*_2_ is another empirical constant.

**Table 2:** Empirical constants used in the one-dimensional mixing model.

Constant	Value	Source
*C*_1_	1/15	Simpson et al. [27]
*C*_2_	0.024	Umlauf [31]
*C*_3_	0.75	Galperin et al. [32], Umlauf [31]

The value of *C*_2_ comes from the formula $c_{\mu }^{\theta }/C_{3}$ as *Ri* → ∞ that can be derived from Eqs (10) and (15) in the cited reference for *C*_2_.

In order to choose an appropriate form for the turbulent length scale, *l*(*z*), we account for the effects of both upper and lower boundaries, and density stratification. Following the study by Simpson et al. [27], the boundary effects were accounted for by choosing *l* = *l*_*H*_(*z*) ≡ *κz*(1 − *z*/*H*), where *κ* = 0.41 is von Karman’s constant. The influence of stratification will be modelled by the methods presented in Umlauf [31] and Jackson et al. [33], which can be applied to shear free stratified mixing situations. In the limit of vanishing shear, and finite stratification, Umlauf [31] has let *l* → *l*_*N*_ ≡ *C*_3_ *k*^1/2^ *N*^−1^, with *C*_3_ an empirical constant (Table 2). This form can be understood in terms of the Ozmidov length scale, defined as *L*_*O*_ ≡ (*ε*/*N*^3^)^1/2^, which is proportional to the largest vertical eddy size in turbulent stratified waters, at large Reynolds numbers. If we associate *l*_*N*_ ∼ *L*_*O*_ and take *ε* ∼ *k*^3/2^/*l* in the above definition, we get the scaling *l*_*N*_ ∼ *q*/*N*. Therefore, we model the turbulent length scale in bounded, stratified waters by the form

$$\begin{array}{c} l^{-1}=l_{H}^{-1}+l_{N}^{-1}\;\Rightarrow \;l=\frac{l_{H}l_{N}}{l_{H}+l_{N}}\;\text{with}\;l_{N}=C_{3}q/\sqrt{2}N. \end{array}$$ (28)

Our 1D mixing model consists of numerically solving Eq (25) to give *q*(*z*), once the forms in Eqs (26)–(28) are substituted. This is then used to calculate *K*(*z*), so that the diffusion equation can be stepped forward in time to give a new *ρ*(*z*, *t*). We use the Newton-Raphson method to solve the energy balance Eq (25), and a semi-implicit finite difference predictor-corrector scheme for the diffusion equation.

**Fig 5:**
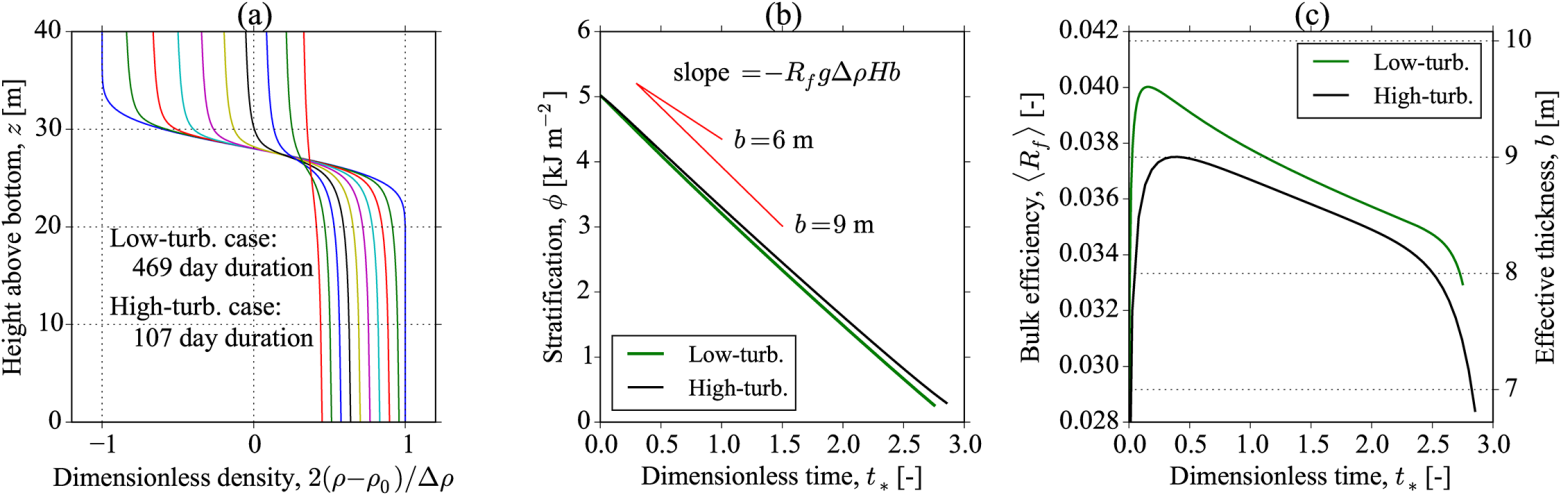
Results of the one-dimensional mixing model 3. (a) The evolution of the dimensionless density profile, 2(*ρ* − *ρ*_0_)/Δ*ρ*, as a complete mixing of the stratification occurs. The results of the low- and high-turbulence simulations show very similar results when the dimensionless time, t_*_ is used. The time to complete mixing is found to be 469 and 107 days for the low- and high-turbulence cases. (b) The evolution of *ϕ* with t_*_ where the red lines indicate the equivalent constant mixing efficiency lines at fixed pycnocline thickness (*b* = 6 and 9 m are shown). (c) Bulk mixing efficiencies 〈*R*_*f*_〉, and the corresponding effective pycnocline thicknesses, *b*. Slight differences in mixing efficiency for the low- and high-turbulence cases are seen.

The results of the modelling are shown in Fig 5, where a number of important features can be seen. First, the evolution of the density profile shows that the pycnocline reaches an approximate equilibrium thickness close to the initial thickness of *b* = 6 m. We would, therefore, expect that the stratification, *ϕ*, should decrease at near constant rate as described in Eq (13). This is indeed what is seen in Fig 5(b), and reflected in the mixing efficiency, 〈*R*_*f*_〉, in Fig 5(c) where after a short initial adjustment it remains at a relatively constant value up to the time of complete mixing at the end of the simulation. Note that as with the previous case 2 mixing model, there is generally a collapse of the data for the low- and high-turbulence simulations if we use the dimensionless time, t_*_. However, slight differences in 〈*R*_*f*_〉 were observed between these cases (Fig 5c).

This behaviour of the pycnocline to preserve its thickness can be seen to be an asymptotic feature of our model with a strongly stratified water column and a constant-in-depth production of turbulence. If the boundary influence on the turbulent length scale is negligible compared to the stratification then *l*_*H*_ ≫ *l*_*N*_ so that *l* → *l*_*N*_. The energy balance leads to the relationship that *q* ∝ *N*^−1/2^. This relation then gives a diffusivity *K* ∝ *N*^−2^, which is just the right vertical dependence so that the pycnocline does not grow in time. This can be understood as the diffusive spreading of the pycnocline being just balanced by the divergence of *K*. In other words, when the pycnocline is strong, and located away from the boundaries, the mixing model predicts that the pycnocline thickness remains relatively constant in time. This lends support for the case 1 mixing model with a constant thickness.

However, despite the relatively constant pycnocline thickness, we find that there is an enhanced rate of mixing compared to the case 1 model with *b* fixed at the initial condition. Assuming that the mixing efficiency, *R*_*f*_ = 0.17, an equivalent value of *b* required to produce the simulated decrease in *ϕ* is found to be approximately *b* = 9 m (Fig 5b, red lines).

Using the above results, we can calculate the appropriate stratification residence time as the time required for complete mixing. This gives a residence time of *τ*_mix_ = 469 and 107 days for the low- and high-turbulence cases, respectively. A summary table of our mixing time scale for all three idealised modelling cases is shown in Table 3.

**Table 3:** Summary of the mixing time scale estimates, *τ*_mix_.

	*τ*_mix_ (days)	
	Low-turb.	High-turb.
Mixing model 1	688	151
2	162	37
3	469	107

## Model Predictions in the Context of Observations

In this section we use a number of available observations and recent measurements in the German Bight region of the North Sea to make inferences as to the potential impact of the wind farm foundation structures on turbulence and mixing. The data sources used are through both measurements made using ocean gliders and fixed moorings, as well as numerical model results.

### Description of data sources

Measurements made from a number of different instruments, and at different times are each described here in turn.

#### Gliders

**Fig 6:**
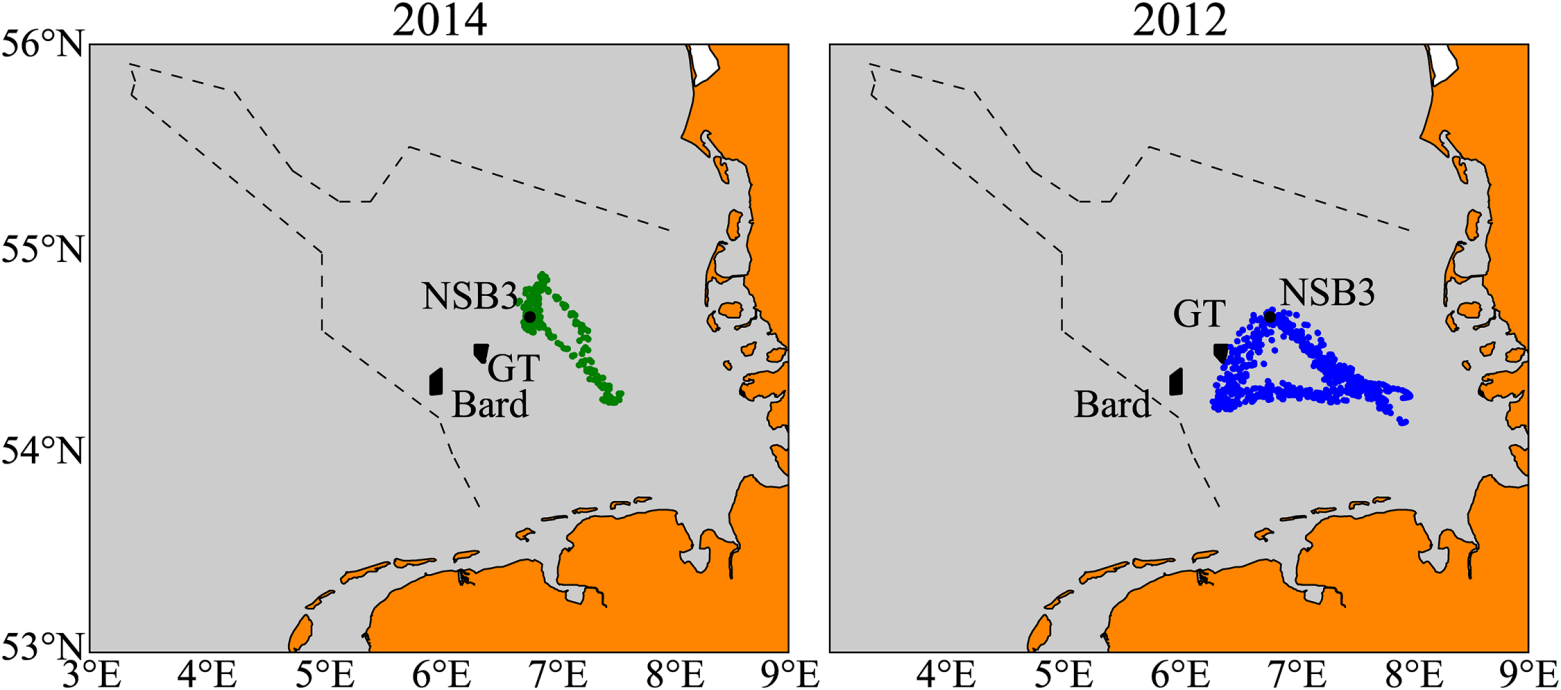
Glider positions for the 2012 and 2014 campaigns.

Field measurements were collected using autonomous ocean gliders [34, 35] during the summers of 2012 and 2014. Gliders adjust their buoyancy by means of an internal pump in order to profile the water column vertically from near the surface to approximately 2 − 3 m above the bed, while using wings to provide a forward momentum. Each glider is equipped with a Seabird CTD to provide measurements of conductivity, temperature and pressure, at a frequency of 0.5 Hz that are used to calculate stratification parameters. The surfacing times of the gliders were approximately 3 hours apart, and allow for the determination of an exact position. These positions for the 2012 and 2014 campaigns are shown in Fig 6.

#### Mooring

A series of temperature and salinity measurements made at the NSB3 station by the BSH (Bundesamt für Seeschifffahrt und Hydrographie) were used to calculate stratification related parameters during the summers of 2004, 2005, 2009-2013. Temperature and conductivity were recorded every hour at the fixed depths of 4, 6, 10, 15, 20, 25, 30, 35 m for temperature, and 6 and 35 m for conductivity. Data was recorded in 40 m total water depth, at a sampling interval of 10 mins, and averaged to produce hourly values. There are significant gaps in the data for many of the sensor depths, and it was only possible to calculate *ϕ*(*t*) for the year of 2009. However, changes in temperature, salinity, and density across the pycnocline were calculated for the other years when available.

#### ADCP

During the summer of 2014, an Acoustic Doppler Current Profiler (ADCP) was also moored on the sea bed close to the NSB3 station from July 16 to August 31. The ADCP was a RDI 600 kHz sampling every 10 minutes using an ensemble of 32 pings. This allowed for the measurement of both components of the horizontal velocity over the depth range of 5 − 38 m in a total water depth of 40 m.

In addition to these measurements, we also use results from the numerical simulation of North Sea stratification in the German Bight.

#### Stratification modelling

Data from simulations carried out and discussed in van Leeuwen et al. [5] will be used to determine the duration of seasonal stratification, and the variability that is present in the German Bight region. These simulations were carried out for a 51 year time period within the North Sea domain (51 to 60°N and −5 to 9°E) using the General Estuarine Transport Model (GETM, see www.getm.eu and [36]). We shall only discuss results of the duration of seasonal stratification at the location 54.5°N, 7.0°E near to the NSB3 measurement station. For full details of the simulations, and validation see van Leeuwen et al. [5].

### Observed stratification

**Fig 7:**
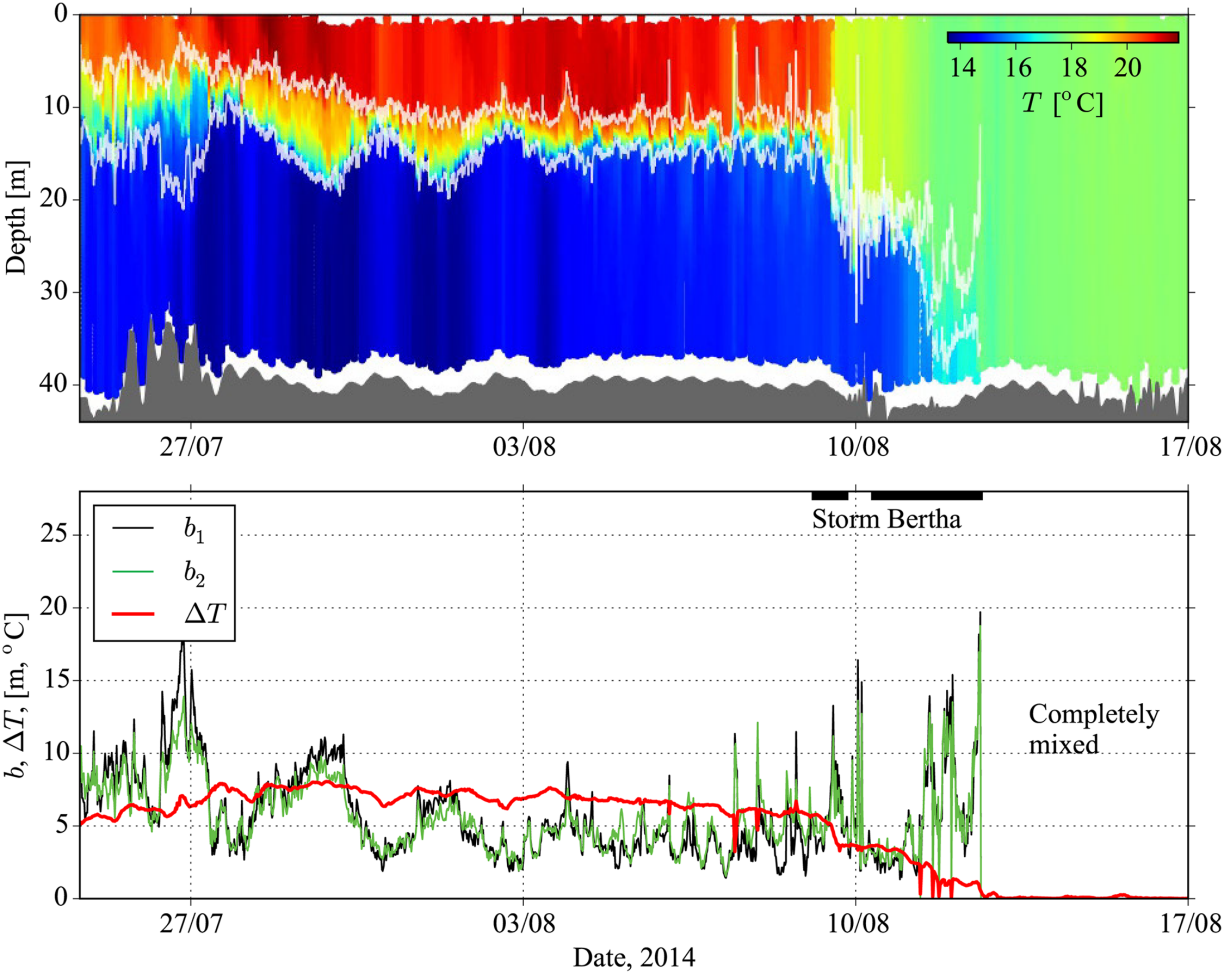
Glider measurements of North Sea stratification, summer 2014. The upper panel shows the conservative temperature in depth and time. The pycnocline region is indicated by the light coloured lines, corresponding to the *b*_1_ definition, and the depth of the sea bed is indicated with dark grey fill. The bottom panel shows thermocline thickness using two different methods (*b*_1_, *b*_2_), and total temperature difference, Δ*T*. The approximate times of Storm Bertha are indicated by the dark bar at the top of the panel, and are defined by times of sustained wind speeds in excess of 10 m s^−1^.

As an example, we begin with an overview of the stratification for July and August 2014 that can be seen in the depth-time plot of water temperature, *T*, shown in Fig 7(a). At the beginning of the measurements the water column has developed a strong stratification with a well-defined pycnocline centred at around 12 m depth. With the arrival of storm Bertha on Aug. 9, persistent winds in excess of 10 m s^−1^ eventually produced a complete mixing of the water column, and an end to the seasonal stratification. This build up of stratification and eventual destruction by fall and late-summer storms is typical of the North Sea seasonal cycle, and will be discussed further below.

A critical factor in determining the bulk mixing efficiency, 〈*R*_*f*_〉, was the thermocline thickness, *b*. This is a quantity that can be readily calculated from the glider CTD measurements. However, exactly how *b* should be defined is not clear, and we are not aware of a generally accepted definition. We shall therefore, use two different definitions with the first, *b*_1_, corresponding to the depth interval over which 80% of the total change in temperature occurs. In the calculation of *b*_1_ we use a sorted temperature profile, T_*_(*z*), obtained by rearranging the measured *T* values in the profile to be monotonically increasing with height. This is done to avoid unrealistic values of *b*_1_ due to the presence of overturns. The second definition, *b*_2_, is based on an integral representation that has previously been used in the study of stratified shear layers [37] given by

$$\begin{array}{c} b_{2}\equiv \int_{0}^{H}\{1-{\left[\frac{2T_{✱}\left(z\right)-\left(T_{u}+T_{l}\right)}{\Delta T}\right]}^{2}\}\;dz, \end{array}$$ (29)

where we define the temperature difference across the thermocline as Δ*T* ≡ *T*_*u*_ − *T*_*l*_ with *T*_*u*_ and *T*_*l*_ the temperatures in the upper and lower layers, respectively. Note that the definition of *b*_2_ has the property that *b*_2_ = Δ*T*/(*dT*/*dz*)_max_, in accord with Eq (17), when T_*_(*z*) has the hyperbolic tangent form.

Time series of thermocline thicknesses *b*_1_, *b*_2_, as well as Δ*T*, measured during the campaign by the glider, are shown in Fig 7(b). Average values of *b*_1_, *b*_2_ over the stratified period, before the arrival of storm Bertha, correspond to 6.0 m and 5.8 m, respectively, and therefore a typical value of *b* = 6 m has been listed in Table 1. Note that if we had defined *b*_1_ to be 90% of the total change in *T* then we find an average value of 8.2 m. We have chosen to use the 80% definition, since it corresponds very closely with the independent definition of *b*_2_. However, this somewhat arbitrary definition of *b* is a limitation of the idealised modelling. It is also not clear what the spatial variability of *b* is in the German Bight, or how it depends on *H*, or other factors.

### Stratification and the seasonal cycle

**Fig 8:**
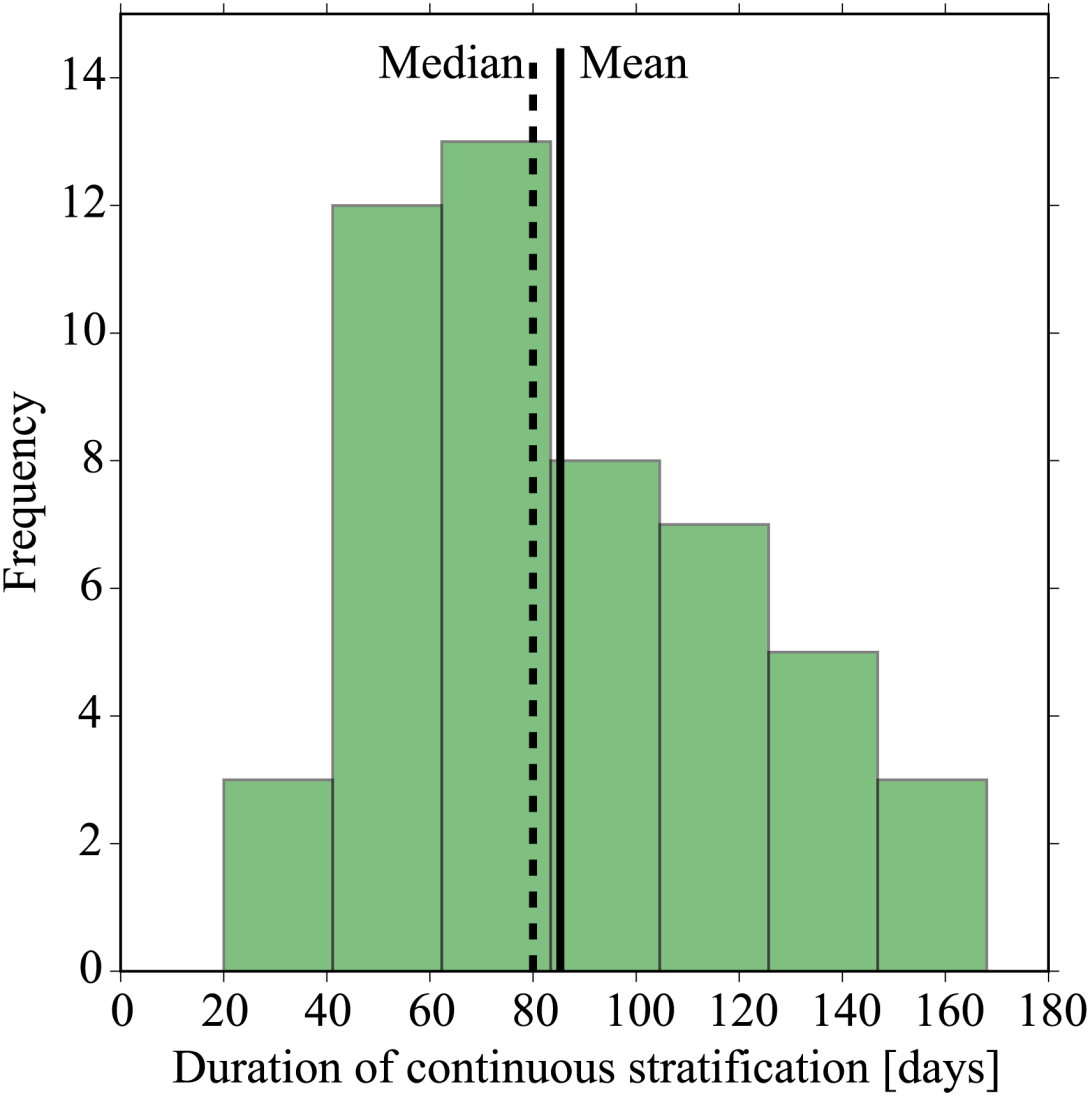
Histogram of the period of longest continuous stratification from the model of van Leeuwen et al. [5]. The data are presented for the location 54.5°N, 7.0°E near to the NSB3 measurement station. Mean and median values for the time of seasonal stratification are 85 and 80 days, respectively. Data supplied by S.M. van Leeuwen, Cefas (UK); contains public sector information licensed under the Open Government Licence v3.0, UK Crown Copyright.

To understand the relevant time scales over which the enhanced OWF-induced turbulence can act to mix the stratification, we must first quantify the typical duration of the seasonal stratification (a second, advective time scale is estimated in the following section). Here we appeal to the North Sea-wide modelling study of van Leeuwen et al. [5], for a representative point close to the NSB3 measurement station. A histogram is plotted in Fig 8 that shows the frequency of the duration of longest continuous stratification for simulations carried out over the years 1958-2008. The figure shows a significant amount of inter-annual variability, with a mean duration of seasonal stratification of 85 days and a standard deviation of 34 days. It will be shown in the following section that this seasonal stratification time scale is not the limiting scale for the mixing of stratification, since advection of stratification through the German Bight is more rapid.

We now consider all dominant processes that are acting to build and destroy the stratification over the course of the seasonal stratification cycle. We begin with a simple depth-integrated model similar to that presented in Simpson and Sharples [1], where an energy balance for the potential energy of the North Sea water column may be written in the general form of

$$\begin{array}{c} \frac{d\phi }{dt}=P_{\text{heat}}-P_{\text{str}}^{✱}-P_{\text{bot}}^{✱}-P_{\text{wind}}^{✱}. \end{array}$$ (30)

The terms on the right hand side (with all $P_{i}^{*}>0$) represent the power input due to atmospheric heating and net incoming solar radiation, and the possible turbulent mixing processes arising from turbine structures, bottom friction, and surface wind stress (including wind waves), respectively, with the asterisk indicating that each must be multiplied by an efficiency.

We evaluate the relative importance of the *P*_str_ term by plotting the observed *ϕ*(*t*) from measurements collected near NSB3. In order to calculate *ϕ*(*t*), we use Eq (7) together with the following form for the water density

$$\begin{array}{c} \rho \left(z\right)=\rho_{u}+\Delta \rho f\left(z\right), \end{array}$$ (31)

where Δ*ρ* ≡ *ρ*_*l*_ − *ρ*_*u*_, and the function *f*(*z*) ≡ [*T*(*z*) − *T*_*u*_]/(*T*_*l*_ − *T*_*u*_). This form assumes that the salinity profile has the same vertical structure as *T*(*z*). We make this assumption because the variation of salinity in the pycnocline is either not known (mooring data, with measurements at only 6 and 35 m), or unrealistic densities are calculated within the pycnocline from the mismatch and time response of the conductivity and temperature sensors (glider data). However, since in each case the upper and lower mixed layer salinity is accurately measured, this contribution to density is accounted for through the Δ*ρ* factor.

**Fig 9:**
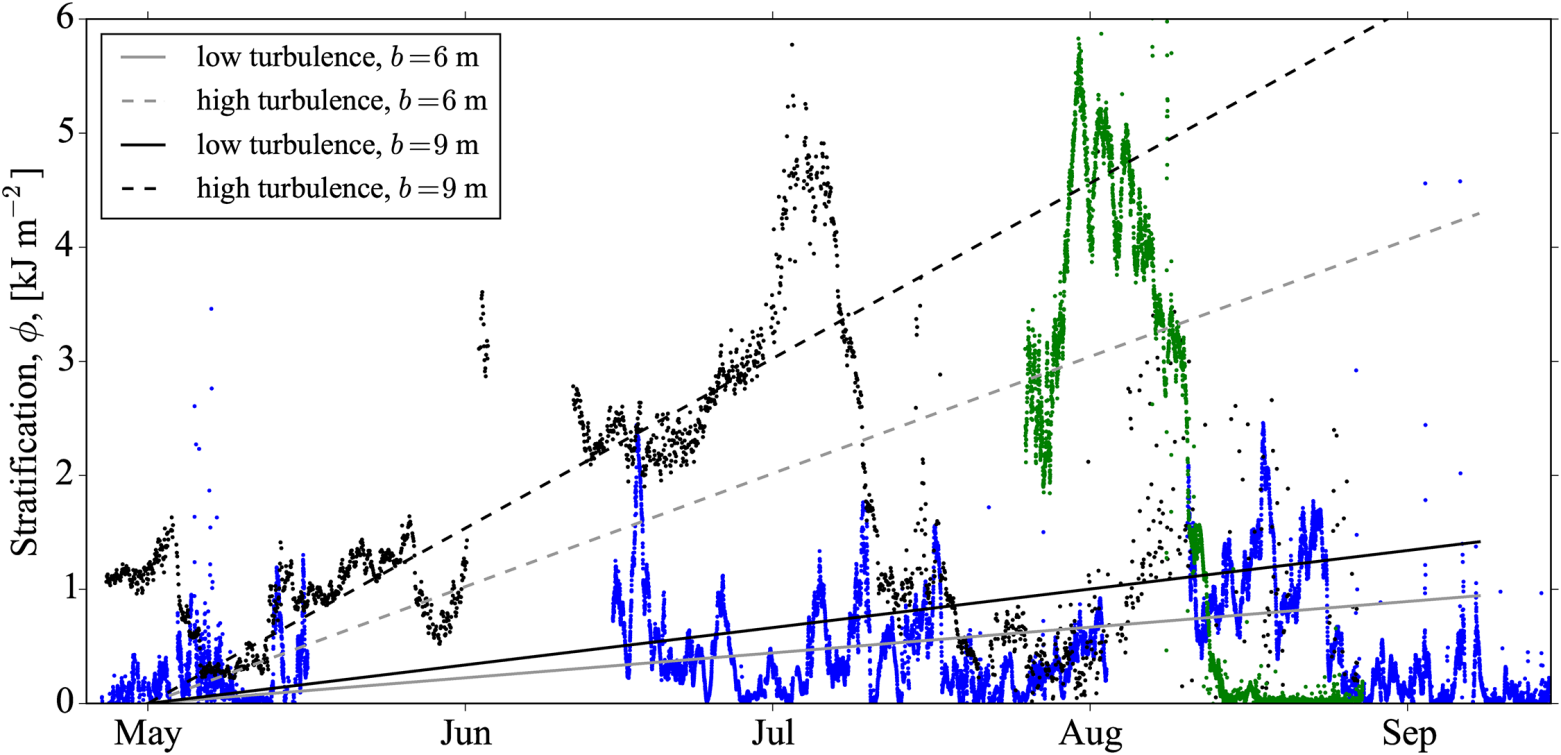
Measurements showing the build up of stratification, *ϕ*, over the summer months (data points) versus the rate of stratification removal by the turbine foundation structures (straight lines). Measured stratification data are from a thermistor mooring at NSB3 in summer 2009 (black points), and glider data collected close to NSB3 from summer 2014 (green points), and from larger scale transects passing through NSB3 in summer 2012 (blue points). None of these stratifications are expected to be affected by OWFs. The lines plot the mixing power of the turbine foundations at two different thermocline thicknesses, *b* = 6, 9 m, based on results from models 1 and 3 using the slope *R*_*f*_ *P*_str_ *b*/*H*, or equivalently the slope 〈*R*_*f*_〉*P*_str_. Also, both the low- and high-turbulence cases are shown with the dashed and solid lines, respectively. All lines are shown starting from an arbitrary initial date of May 1. Mooring data from 2009 were obtained from BSH.

At these times, and in the measurement locations, we expect very little influence from OWFs that could produce a *P*_str_ contribution. We can therefore estimate the relative size of *P*_str_ by overlaying our previously determined estimate based on a fixed width thermocline. In other words, in Fig 9 we can compare the rate of growth of stratification (*dϕ*/*dt*) with the rate of removal by the turbine structures, i.e.,

$$\begin{array}{c} \frac{d\phi }{dt}=\left(P_{\text{heat}}-P_{\text{bot}}^{✱}-P_{\text{wind}}^{✱}\right)\;\text{vs}.\;P_{\text{str}}^{✱}\equiv \left\langle R_{f}\right\rangle P_{\text{str}}. \end{array}$$ (32)

The left equation is given by the observations and the 〈*R*_*f*_〉*P*_str_ term represented by the various lines depending on the *b*, *C*_*D*_, and foundation type chosen, i.e., the different bulk mixing efficiencies 〈*R*_*f*_〉 = *R*_*f*_ *b*/*H* and low- and high-turbulence cases. Fig 9 suggests that the mixing due to wind farm turbulence could represent a significant contribution to the overall potential energy budget of the stratification.

**Fig 10:**
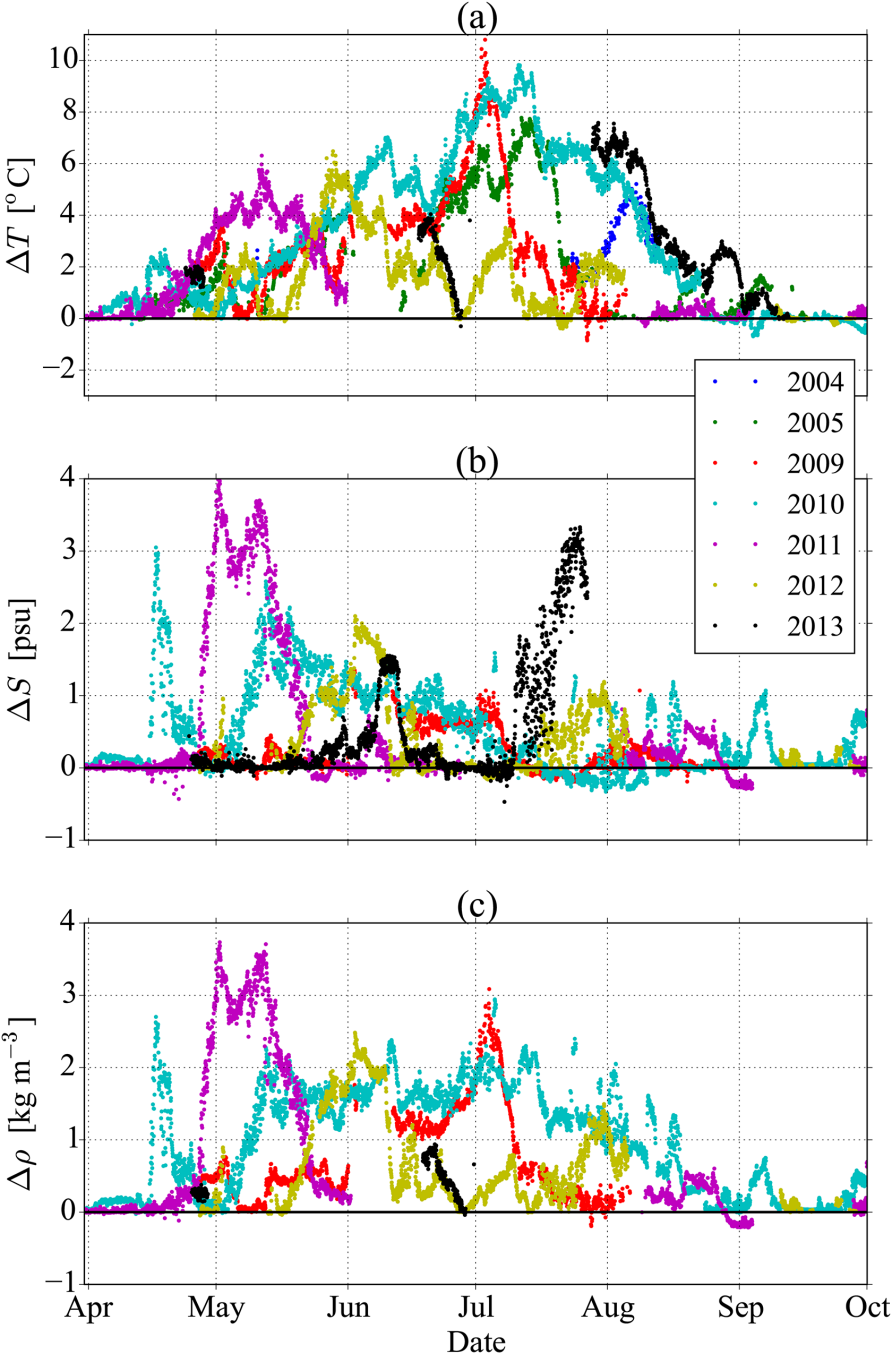
Measurements showing the difference in (a) temperature, (b) salinity, and (c) density across the pycnocline over the summer months at NSB3. These differences were calculated between measurements in the surface layer at 4 or 6 m deep (depending on the availability of data) and at depths of 35 m in the lower layer. Data were obtained from BSH.

As shown in Fig 9, *ϕ* gradually increases in the early summer months as the *P*_heat_ term increases relative to the other stratification removing terms. However, there is significant variability of the seasonal peak in stratification, as seen in the glider measurements from 2012. Based on the measurements in 2009, and 2014, we can take a typical value for the peak stratification as *ϕ*_max_ = 5.0 kJ m^−2^(Table 1). In order to provide a greater justification for taking this representative value based on only two seasons, we analyse other years from the mooring data at NSB3 (Fig 10). The data from these years have captured the total change in temperature and salinity (and therefore also density) across the pycnocline, but do not have the vertical resolution required for computing *ϕ*. Assuming the error function form for the density profile, as described in mixing model 2, as well as the other typical values for *b* and *h* listed in Table 1, this representative value of *ϕ*_max_ = 5.0 kJ m^−2^ corresponds to a density difference of Δ*ρ* = 3.1 kg m^−3^. This value of Δ*ρ* is also found to be the average peak measured from the data in years 2009-2012 (Fig 10), supporting our choice of *ϕ*_max_ = 5.0 kJ m^−2^. The measurements from 2004 and 2005, plotted in Fig 10, unfortunately do not resolve the peak in summer stratification, and have therefore not been used to calculate the average peak Δ*ρ*.

It should be noted that the salinity contribution to Δ*ρ* is often large and highly variable (Fig 10b). This is expected to be the influence of the freshwater plume from the Elbe River that flows along the eastern coastline of Germany and Denmark. This freshwater is expected to have less of an influence in the German Bight further away from the coasts, and may lead to lower values of Δ*ρ* and *ϕ* there. This importance of freshwater influence was also noted in van Leeuwen et al. [5], where they found that conditions near NSB3 were classified as a region of freshwater influence 12% of the time.

## Accounting for Advection and Wind Farm Size

Thus far our model analysis has assumed that the wind farms occupy the entire sea, so that there is effectively no advection of stratification through the wind farm area. However, it is known that there is a residual mean flow, superimposed on the tidal motions, that is responsible for an advection of the water column through the North Sea [38]. When the wind farm occupies a finite extent then the mixed water will eventually be replenished with fresh stratification from outside, and the time scale for this replenishment is denoted by *τ*_adv_. This effect was ignored in the previous sections in order to estimate a mixing time scale, and time scale for the seasonal stratification, which are both independent of the wind farm size.

**Fig 11:**
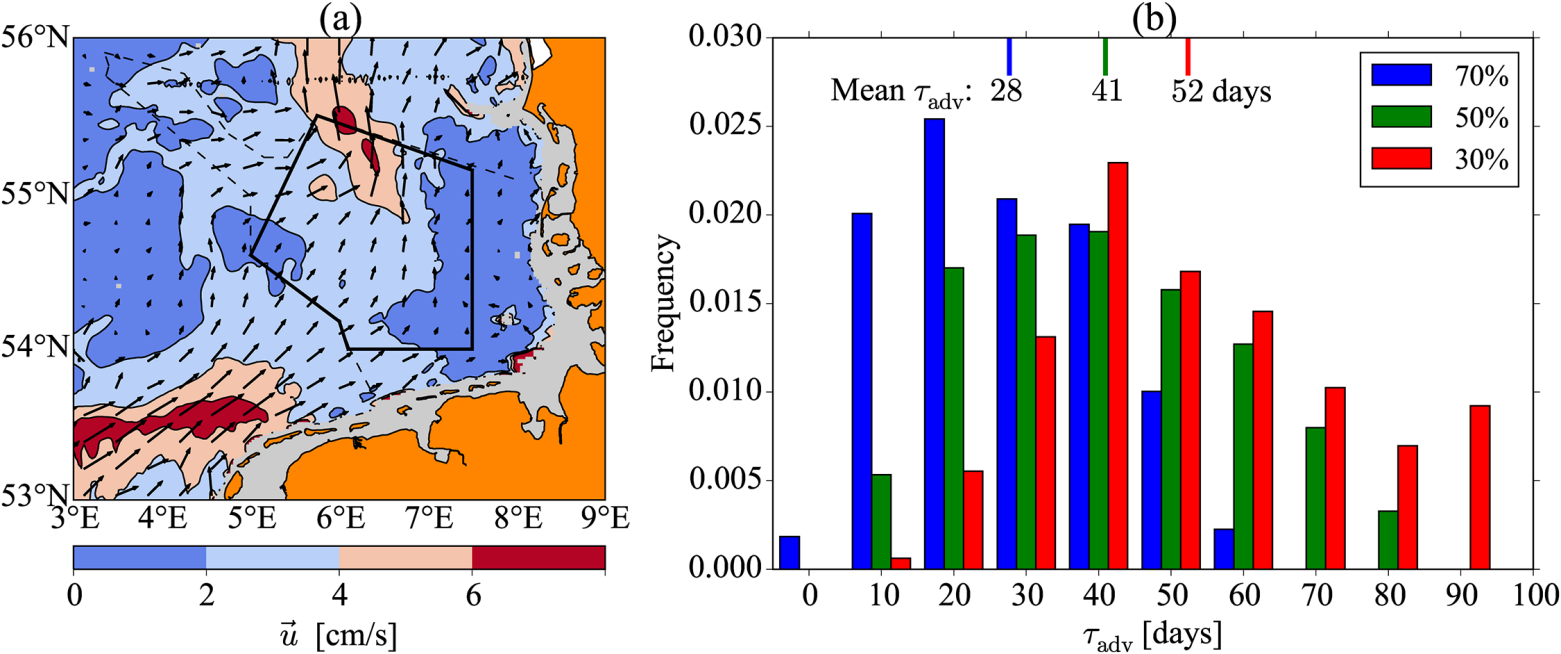
Residual currents. (a) Map of residual (depth-averaged) currents in the German Bight with contours indicating the magnitude, and arrows indicating direction and magnitude. The averaging period is taken over all years between 1958-2014. The dark outlined area represents the study region referred to in the text. (b) Histograms showing the distribution of times (*τ*_adv_) that correspond to the minimum residence time within the study area of 70, 50, and 30% of the drifters. The mean values of each distribution are indicated by the lines at the top, and bin edges are at *τ*_adv_ = −5, 5, 15, 25, ….

What is relevant for our analysis is the amount of time a water column is expected to spend within the elevated mixing region of the wind farm. This advective time scale is determined by both the length of the farm, and the strength of the mean circulation. Two scenarios will be examined: (i) OWFs on the scale of current construction (e.g., the Bard and Global Tech farms) with a typical length of *L* = 8 km, and (ii) a future scenario where OWFs fill the study area shown in Fig 11(a) with a length scale of *L* ≈ 100 km. These two scenarios will allow for impacts that span the full range of OWF development options in the German Bight.

Residual currents are estimated from the TRIM model [22], as previously described in the tidal current estimates section. To get a picture of the mean (depth averaged) circulation through the German Bight region, we have used an averaging period running over the course of the entire simulation length, from years 1958 to Aug. 2015 (freely available from doi: 10.1594/WDCC/TRIM-NP-2d-PCA_ResCurr). The result is shown in Fig 11(a), and shows that a bulk advection that is towards the north-east through the proposed OWF development area.

To estimate a representative *τ*_adv_ for the relatively small-scale (*L* = 8 km) OWFs currently built, we make the assumption that the spatial gradients of the residual current do not vary appreciably over the length of the OWF. By inspection of Fig 11(a), this appears to be a good assumption, and allows us to write $\tau_{\text{adv}}=L/\bar{u}$, where $\bar{u}$ refers to the time-averaged residual current magnitude. Using the representative range of mean residual current speeds in the study region of $1<\bar{u}<6$ cm s^−1^, and *L* = 8 km, gives 1.5 < *τ*_adv_ < 9.3 days as a typical range in the German Bight. Specifically, for the Bard and Global Tech OWFs we find $\bar{u}=2.3,2.1$ cm s^−1^, and therefore, *τ*_adv_ = 4.0, 4.4 days, respectively.

When considering mean advection speeds through larger regions where significant spatial gradients exist, it is not possible to use a simple spatial average for the calculation of mean residence times. This is due to the Lagrangian nature of particle advection, since particles spend more time in regions of lower velocities than in regions of larger velocities. This makes the computation of *τ*_adv_ more complicated for the large-scale OWF development scenario. The procedure that we take to estimate *τ*_adv_ is as follows. First, the study region indicated in Fig 11(a) is seeded with 2,000 evenly distributed Lagrangian drifters. The position of each drifter is integrated over a time period of 90 days, and the total duration that the drifter spends in the study region is calculated. This is done with an integration time beginning on each separate day from May 1 until July 1, sufficient to cover the summer stratification season when the 90 day integration time is included, for the years 2001-2005, 2009, 2012, 2014 (largely chosen to match with years in which stratification data is available). Each 90 day integration is treated as an independent estimate of the distribution of time values from each drifter, which is the duration that the drifter spends in the study area, allowing for exit and reentry. The minimum times recorded by 70, 50, and 30% of the drifters for each distribution are then calculated, giving three different estimates of *τ*_adv_, and their subsequent distribution over all experiments is shown in Fig 11(b). In other words, for a single integration experiment with 2,000 drifters, an estimate of *τ*_adv_ represents the minimum time spent in the study area of 70% of the drifters in the case of the blue histogram. By calculating means of these *τ*_adv_ distributions we arrive at three estimates of *τ*_adv_ = 28, 41, 52 days, that correspond to the averaged minimum residence time within the study area of 70, 50, and 30% of the drifters, respectively. Note that if any one of the years that drifter simulations were performed are excluded from the analysis, the mean values of *τ*_adv_ change by no more than ±3 days.

This analysis shows that the advective time scale provides the greatest control over the total amount of mixing, since it is generally much shorter than the time scale of the seasonal stratification (∼85 days), and also the majority of the mixing time scales presented in Table 3. However, there is a very large variability that is present in these distributions, and it can be seen that the difference in the means of the 70, 50, and 30% values of *τ*_adv_ are smaller than the variability of a single distribution. In the next section we provide estimates of the reduction in stratification that these time scales imply.

## Discussion

### Impacts on the large-scale stratification

As the main results of this study we have estimated both the mixing and advective time scales, *τ*_mix_ and *τ*_adv_, and compared them to the observed rate of build up of stratification, *dϕ*/*dt*. It is interesting now to relate these two time scales by looking at the case of a wind farm of finite size in a steady residual current with all other stratification removing and building processes neglected, i.e., assuming a balance between advection and wind farm mixing. In formulating such a model, we make the assumption that a representative advection speed through the farm is given by *L*/*τ*_adv_. Then, if the stratification entering the wind farm is *ϕ*_0_ = *ϕ*(*x* = 0), the total change in stratification after passing through the farm, Δ*ϕ* ≡ *ϕ*_0_ − *ϕ*(*x* = *L*), is given by 〈*R*_*f*_〉*P*_str_ *τ*_adv_. In this case, the fractional reduction of stratification can be represented by the ratio of our two fundamental time scales, i.e.,

$$\begin{array}{c} \frac{\Delta \phi }{\phi_{0}}=\frac{\tau_{\text{adv}}}{\tau_{\text{mix}}}, \end{array}$$ (33)

where here *τ*_mix_ ≡ *ϕ*_0_/〈*R*_*f*_〉*P*_str_ is the time scale to mix *ϕ*_0_. This shows explicitly that the impact of the OWF structures on the stratification is dependent on the ratio of the advective and mixing time scales. Note that we could have also formulated this model in a Lagrangian perspective in terms of the time spent by a drifter within the elevated mixing region of the OWF; the result is the same. This idealised model can be used as a very rough guide to judge the spatial extent to which OWFs can be built before significant impacts to the stratification are expected.

Table 4 shows a summary of the reduction in stratification that will occur through OWFs from our two scenarios of (i) existing farms where *L* ≈ 8 km, and (ii) for the study region of Fig 11(a) with *L* ≈ 100 km. This is done also for the three different mixing models, for both low- and high-turbulence conditions, and for the three different drifter coverages of 70, 50, and 30%. In computing *τ*_mix_ for the large farm case in Table 4, we have used a *P*_str_ that is obtained from a spatial average over the study area, rather than the values typical of the Bard and Global Tech farms listed in Table 1. These values were found to be 2.8 and 13 mW m^−2^ for the low- and high-turbulence cases, close to that found for the Bard and Global Tech farms. The difference in the reduction of stratification from the 70 and 30% drifter coverages is approximately a factor of two, and the inter-annual variability of *τ*_adv_ can be up to a factor of 4 or more. This shows that the impact of the OWF-induced mixing is sensitive to the large variability of the advection by residual currents through the German Bight, which is largely driven by the variability in wind forcing.

**Table 4:** Estimates of the reduction in stratification Δ*ϕ*/*ϕ*_0_.

	Small farm (*L* ≈ 8 km)		Large farm (*L* ≈ 100 km)					
			Low-turb.			High-turb.		
Mixing model	Low-turb.	High-turb.	70%	50%	30%	70%	50%	30%
1	0.01	0.03	0.03	0.05	0.06	0.16	0.23	0.30
2	0.02	0.12	0.14	0.20	0.25	0.65	0.95	>1
3	0.01	0.04	0.05	0.07	0.09	0.24	0.35	0.45

Summary of the results for the three different mixing models, two OWF length scales, and for both the low- and high-turbulence cases. Values of Δ*ϕ*/*ϕ*_0_ > 1 represent a complete mixing. The percentages refer to the drifter coverages of mean *τ*_adv_ values.

Note that the computed reduction in stratification assumes that no stratification building processes are acting. If we were to include these then we could modify Eq (33) by adding the term −*τ*_adv_/*τ*_*s*_ to the right hand side (with a negative sign representing the addition of stratification), where *τ*_*s*_ is a typical time scale for the stratification to build up to the level *ϕ*_0_, i.e., the seasonal stratification time scale. In addition, we have made the assumption that there are no OWFs upstream so that the water column entering the elevated mixing region of the OWFs is “pristine”. The results indicate that the high-turbulence scenario with extensive OWF development in the German Bight (*L* = 100 km) could significantly impact the large-scale stratification, whereas current construction levels have only a very small impact.

### Assumptions and uncertainties

Throughout this study we have made the assumption that the mixing efficiency, expressed as a flux Richardson number *R*_*f*_, has the constant value of 0.17. Despite this being common practise in oceanographic mixing studies [28], there has also been much evidence that the efficiency is not constant, and depends on the particular mixing process [39]. In the particular case of mixing driven by the OWF structures, it is likely that the turbulence is confined to a narrow wake region trailing the structure where intense mixing occurs [40]. This would suggest that there is the possibility of much lower mixing efficiencies than the standard value of *R*_*f*_ = 0.17. This is due to the fact that the turbulence could rapidly mix the stratification in the narrow wake before it has been fully dissipated by viscous friction—essentially expending work to mix already mixed fluid. If this is the case, which is at least expected under conditions of weak stratification (to be defined shortly), then our mixing models could begin to break down since the density structure of the water column behind the structures will no longer resemble the background profiles that we have assumed.

A rough guide to measure the potential disturbance of the density structure of the wake could be the dimensionless number *Π* ≡ *ρ*_0_ *C*_*D*_ *HU*^2^/2*ϕ*, where all variables are as previously defined, and *U* is a tidal current velocity scale. This number expresses the ratio of turbulent kinetic energy per unit volume to potential energy per unit volume of the stratification, in the wake. Using typical values of *ρ*_0_ = 1026 kg m^−3^, *U* = 0.3 m s^−1^, *C*_*D*_ = 0.3, *H* = 40 m, and *ϕ* = 5 kJ m^−2^, gives a value of *Π* = 0.1, whereas if we now take a larger drag coefficient *C*_*D*_ = 1.0 and weaker stratification *ϕ* = 1.8 kJ m^−2^, the ratio of the energies is of order *Π* = 1. This rough argument suggests that the wake region could span both regimes of a relatively intact density field at low *Π*, and a mixed wake for Π≳1. In this mixed wake case, additional horizontal processes will be responsible for the mixing and lateral adjustment of the wake to a state of stable minimum potential energy that are not considered in this study.

Another fundamental assumption made in this initial study is the neglect of feedbacks between OWFs and the natural turbulence and mixing processes of the North Sea. In reality we expect there to be an interaction between the OWF induced mixing of the pycnocline with the highly turbulent upper mixed layer and bottom boundary layer. This type of modelling could be carried out with a more sophisticated turbulence model than presented, however, a major limiting factor is the difficulty in arriving at an accurate representation of natural mixing processes operating within the strongly stratified pycnocline [2]. Furthermore, the laboratory experiments of Whitehead [29] show that, even in an idealised setting, the mixing of a pycnocline by a circular cylinder can exhibit complicated physics, which in some instances can lead to the formation of a layered stratification. The prediction of such “shock” solutions poses an extremely difficult task for any sufficiently general parameterisation of turbulent mixing.

The idealised modelling presented here has identified a number of important uncertainties that significantly affect the predicted mixing levels. The two that have been found to be the most important are (i) uncertainty in the OWF structure drag coefficient and foundation type, and (ii) the uncertain evolution of the pycnocline thickness. Whereas the evolution of stratified (pycnocline) mixing is still a subject of ongoing research, drag coefficients are generally well tabulated for smooth circular cylinders. The uncertainty arises, however, due to both the effects of upstream turbulence, as well as the surface roughness. Although generally designed to be smooth circular cylinders, OWF foundations have been found to be sites of abundant mussel growth and biofouling [14, 40]. This growth would be expected to increase the relative roughness of the structure thus altering the drag coefficient in time. It is also possible that scour protection is used to protect the foundation structures. This would also be expected to increase the drag coefficient, however, no details on this have been obtained.

This discussion of uncertainties applies only to our chosen problem of estimating impacts of OWF structure-induced turbulence and mixing, and we have not considered other possible physical effects such as changes in wind forcing [19, 41–43], or the alteration of tidal currents [18].

## Conclusions

With the large-scale planning and construction of offshore wind farms (OWFs) in coastal seas that are deep enough to support the development of seasonal stratification, we have provided a first, order-of-magnitude estimate of the potential impact this development may have on the mixing of stratification. This mixing is induced by the turbulent wake of the OWF foundation structures as the tidal currents continuously move past. Using idealised modelling we have developed a series of estimates of the mixing time scale that characterises the time to complete mixing. This time scale was generally found to be larger, though comparable to, the summer stratification period, showing that the estimated mixing could be important for the development of stratification. However, these estimates were found to be sensitive to both the time evolution of the pycnocline thickness and the drag of the foundation structures—both of which are uncertain. In addition, estimates of the advective time scale, the duration a water parcel is likely to spend inside the enhanced mixing region of a wind farm of finite size, show that, for a significant impact on the stratification, extensive regions of the North Sea must be covered in OWFs. At the current construction levels (Fig 1) we should not expect any large-scale changes to the stratification of the North Sea. However, in future development scenarios where OWFs fill large portions of the German Bight (Figs 1 and 11a) we could expect significant reductions in the stratification. These impacts are expected to be highly variable due to the dependence of the advective time scale on wind forcing and its variability.

Given that a significant reduction in stratification could be possible from large-scale OWF development, it is still uncertain whether this impact could have positive or negative effects (see introduction and [10]). However, one possible mitigation strategy could involve the use of floating or semi-submerged OWF platforms [44] in order to minimise the effects.

Future work on the impact of OWFs on the mixing of stratification could focus on a number of different directions. Firstly, it is necessary to better understand the local turbulence production and induced mixing of the different OWF foundation structures so that more accurate mixing parameterisations can be developed. Second, these parameterisations could then be used in larger scale regional models to conduct ensemble averages over many seasons, thus providing a statistical measure of the spatial changes for given OWF development scenarios. Finally, it is also important to understand the effects of the enhanced mixing on the scale of an individual farm as they currently exist, i.e., approximately 10—20 km. Over this scale the enhanced mixing could show cascading effects on nutrient levels, ecosystems, and marine mammals. This is due to the strong interaction between turbulence levels and the growth of phytoplankton in general [45], as well as in the North Sea in particular [46]. The physical-biological interactions in OWFs are particularly unknown [19], and it is important to quantify these effects in order to have a complete understanding of the true impact of offshore wind farms.

## Supporting Information

S1 File Wind farm foundation structure parameter estimates. (PDF)
