# Supplementary material for: Potential Impacts of Offshore Wind Farms on North Sea Stratification
Source: PLoS One. 2016 Aug 11;11(8):e0160830. doi: 10.1371/journal.pone.0160830 (PMC4981390; doi:10.1371/journal.pone.0160830)
Supplement: S1 File — (PDF) [file pone.0160830.s001.pdf]

S1 Appendix

J.R. Carpenter, L. Merckelbach, U. Callies, S. Clark, L. Gaslikova, B. Baschek

Institute of Coastal Research, Helmholtz Zentrum Geesthacht

S1 Details of wind farm foundation structure parameter estimates

Bard 1. The foundation structures at Bard 1 are of the tripile type, consisting of three circular cylinders of diameter $D = 3.35$ m, and a mean spacing of 866 m [3]. The frontal area, A, of the wind farm foundation structure can be easily calculated using $A = 3DH$, giving $A = 404$ m^2. We have neglected any armouring that is placed around the piles on the sea bed as erosion protection, and assumed that all three piles contribute to the frontal area, i.e., there is no sheltering effect.

Global Tech 1. Information concerning the exact dimensions of the tripod structures in the Global Tech 1 farm has not, to our knowledge, been made public, and was unknown during the writing of this study. We have, however, estimated the frontal area through a schematic found online [1]. Using the few dimensions that have been marked on the schematic we have estimated a scale ratio for the figure (1:400), and used this to measure the lengths and diameters of the individual cylindrical sections. A number of the sections are tapered and in this case we take a representative diameter consisting of the average of the two end diameters. We approximate the frontal area by the following formula

$$A = D_1L_1 + D_2L_2 + 2[\cos(\theta)D_3L_3 + D_4L_4 + \cos(\theta)D_5L_5], \quad (1)$$

where the lengths L_i and the diameters D_i are labelled in Fig. S1, and given in Table S1. The angle, θ, between the foundation feet and a frontal plane is taken as 30°. We do not consider the third cylinder group in the lee of the diagram for the frontal area since it is at least partially sheltered from the oncoming flow. The mean spacing is found from the number of turbines (80) and the area of the farm (43 km^2) from [4].

S1 Figure

Rough sketch of the tripod foundation structure found at Global Tech 1. The numerical labels refer to the different cylindrical sections that we have used to estimate the frontal area. The approximate lengths of the cylindrical sections are indicated by the dots, and are estimated from scaling the diagram found in [1]. The rear group of cylinders in the third leg is not shown. This diagram has been modified from the original, and is for illustrative purposes only. A more accurate depiction can be found in [2].

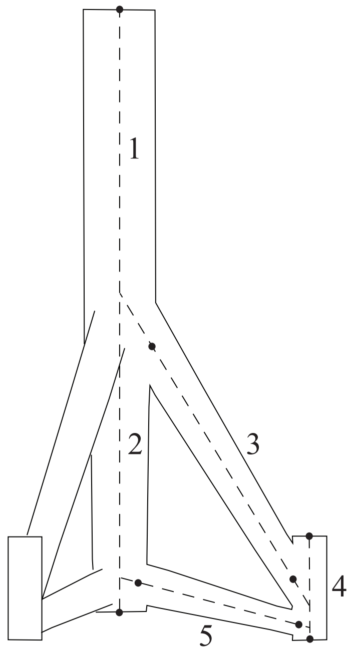

S1 Table

Table 1: Lengths and diameters in the estimation of Global Tech 1 tripod frontal area.

Length/Diameter	Value (m)
D_1	6.4
D_2	$5.6^{\dagger}$
D_3	$3.6^{\dagger}$
D_4	2.9
D_5	$2.9^{\dagger}$
L_1	12.4
L_2	28.8
L_3	31.6
L_4	9.7
L_5	14.0

†Obtained from averages of two cross-sections.

References

[1] L. Siemers. Global tech 1 offshore wind farm logistics and installation, 2013. URL http://http://www.nautischer-verein-bremerhaven.de/downloads/Schifffahrt_und_Umwelt/2013/HTS%20Siemers%20NVBHV%20Schiffumwelt%2020130912.pdf. Retrieved 09 November 2015.

[2] S. Thöns, M.H. Faber, and W. Rücker. Ultimate limit state model basis for assessment of offshore wind energy converters. *J. Offshore Mech. Arct. Eng*, 134(3):031904, 2012. doi: 10.1115/1.4004513.

[3] www.4coffshore.com, 2015. URL http://http://www.4coffshore.com/windfarms/contracts-on-bard-offshore-1-de23.html. Retrieved 09 November 2015.

[4] www.4coffshore.com, 2015. URL http://www.4coffshore.com/windfarms/global-tech-i-germany-de09.html. Retrieved 09 November 2015.
